# Association of Hepatitis C Virus Replication with the Catecholamine Biosynthetic Pathway

**DOI:** 10.3390/v13112139

**Published:** 2021-10-23

**Authors:** George Mpekoulis, Vassilina Tsopela, Georgios Panos, Vasileiοs Siozos, Katerina I. Kalliampakou, Efseveia Frakolaki, Constantinos D. Sideris, Alice G. Vassiliou, Diamantis C. Sideris, Dido Vassilacopoulou, Niki Vassilaki

**Affiliations:** 1 Laboratory of Molecular Virology, Hellenic Pasteur Institute, 11521 Athens, Greece; g.mpekoulis@pasteur.gr (G.M.); vas.tsopela@gmail.com (V.T.); panos.georgios.bio@gmail.com (G.P.); basilis.sz@hotmail.com (V.S.); kalliamp@yahoo.gr (K.I.K.); sevif@pasteur.gr (E.F.); con97sider@gmail.com (C.D.S.); 2 GP Livanos and M Simou Laboratories, 1st Department of Critical Care Medicine & Pulmonary Services, School of Medicine, National and Kapodistrian University of Athens, Evangelismos Hospital, 10676 Athens, Greece; alvass75@gmail.com; 3 Section of Biochemistry and Molecular Biology, Faculty of Biology, National and Kapodistrian University of Athens, 15701 Athens, Greece; dsideris@biol.uoa.gr (D.C.S.); didovass@biol.uoa.gr (D.V.)

**Keywords:** Hepatitis C virus, viral replication, catecholamine biosynthetic/metabolic pathway, L-Dopa decarboxylase, tyrosine hydroxylase, dopamine beta-hydroxylase, monoamine oxidase

## Abstract

A bidirectional negative relationship between Hepatitis C virus (HCV) replication and gene expression of the catecholamine biosynthetic enzyme L-Dopa decarboxylase (DDC) was previously shown in the liver and attributed at least to an association of DDC with phosphatidylinositol 3-kinase (PI3K). Here, we report that the biosynthesis and uptake of catecholamines restrict HCV replication in hepatocytes, while HCV has developed ways to reduce catecholamine production. By employing gene silencing, chemical inhibition or induction of the catecholamine biosynthetic and metabolic enzymes and transporters, and by applying the substrates or the products of the respective enzymes, we unravel the role of the different steps of the pathway in viral infection. We also provide evidence that the effect of catecholamines on HCV is strongly related with oxidative stress that is generated by their autoxidation in the cytosol, while antioxidants or treatments that lower cytosolic catecholamine levels positively affect the virus. To counteract the effect of catecholamines, HCV, apart from the already reported effects on DDC, causes the down-regulation of tyrosine hydroxylase that encodes the rate-limiting enzyme of catecholamine biosynthesis and suppresses dopamine beta-hydroxylase mRNA and protein amounts, while increasing the catecholamine degradation enzyme monoamine oxidase. Moreover, the NS4B viral protein is implicated in the effect of HCV on the ratio of the ~50 kDa DDC monomer and a ~120 kDa DDC complex, while the NS5A protein has a negative effect on total DDC protein levels.

## 1. Introduction

Hepatitis C virus (HCV) infection, with ~71 million cases worldwide, causes chronic liver injury that could eventually lead to liver cirrhosis and hepatocellular carcinoma (HCC) [1]. The global epidemic of HCV is distributed unevenly, with the Eastern Mediterranean region and Europe having the highest prevalence [1,2,3]. The HCV genome, a positive sense, single-stranded RNA, encodes a single polyprotein of ~3000 amino acids [4,5]. This is proteolytically cleaved into structural proteins (core, E1 and E2) that form the viral capsid, p7, which is necessary for the assembly and release of infectious virions, and non-structural (NS) proteins (NS2, NS3, NS4A, NS4B, NS5A and NS5B), orchestrating HCV replication in endoplasmic reticulum (ER) membrane protrusions [6].

We have previously reported an emerging bidirectional relationship between HCV RNA replication and L-Dopa decarboxylase (DDC) [7], an enzyme that catalyzes the biosynthesis of bioactive monoamines [8]. Such are the catecholamine dopamine (DA) and the indolamine serotonin/5-hydroxytryptamine (5-HT), produced by decarboxylation of L-3,4-dihydroxyphenylalanine (L-Dopa) and L-5-hydroxytryptophan (5-HTP), respectively. Although DDC was originally studied for its involvement in neurotransmission, it is also expressed and active in a variety of peripheral organs, including the liver [9], and has an additional physiological role in cell proliferation and apoptosis [10,11]. It has been suggested that the latter function is mediated by the physical and functional interaction of DDC with phosphatidylinositol 3-kinase (PI3K) that inhibits the kinase [11]. Concerning HCV infection, our previous data have shown a negative correlation between *DDC* mRNA and viral RNA levels in the liver samples of chronically infected patients [7]. Moreover, in cultured hepatocytes, DDC mRNA and protein levels were down-regulated by HCV RNA replication, at least through PI3K. In turn, viral replication was reduced by DDC overexpression and favored by the inhibition of DDC activity [7].

The possible association of HCV infection with components of the catecholamine biosynthesis and metabolism pathway other than DDC has not been addressed. The catecholamines, dopamine, norepinephrine (NE) and epinephrine (EN) are derived from tyrosine via a series of enzymatic conversions [12]. Tyrosine is catalyzed to L-Dopa by tyrosine hydroxylase (TH). Subsequently, DA is synthesized from L-Dopa by DDC and is transferred from the cytosol into vesicles by the vesicular monoamine transporter (VMAT). There, it is either stored or converted into NE by the membrane-bound dopamine-beta-hydroxylase (DBH). Fusion of the catecholamine-containing vesicles with the cell plasma membrane releases their content from the cell. VMAT is also important for the uptake of catecholamines by the cell through the plasma membrane catecholamine transporters. In the cytosol, NE is transformed to EN by the activity of norepinephrine (phenylethanolamine) N-methyltransferase (PNMT). The catecholamines and serotonin are autoxidized in the cytosol. Their auto-oxidation can be limited by monoamine oxidases (MAO) that catalyze their oxidative deamination [12,13]. Catecholamines are protected from oxidation by their VMAT-dependent storage in vesicles, which have acidic pH [14].

Expression of the catecholamine pathway associated enzymes is not restricted to neuronal cells but has also been detected in non-neuronal tissues [12]. Specifically, TH and DBH expression has been reported in peripheral organs [15] and in the liver [16], respectively. PNMT activity is not detectable in the liver, where EN is synthesized by a less specific N-methyltransferase [17]. Concerning VMAT, among the two known isoforms, only VMAT2 is expressed in the liver [18,19]. Moreover, the two MAO isoforms, MAO-A and MAO-B that have differences in substrate specificity and inhibitor selectivity [20], are expressed in the majority of peripheral tissues, including the liver [21,22]. MAO-B expression and activity have also been shown in human hepatoma cell lines [23,24]. Finally, in hepatocytes, the low-affinity organic cation transporters (OCTs) have an important role in the uptake of catecholamines [25,26], whereas the high-affinity classical transporters of dopamine (DAT), serotonin (SERT) and norepinephrine (NET) that function in the nervous system, are not expressed in these cells [27].

Dopamine produced in the periphery has been reported to influence cellular metabolism, proliferation and apoptosis in the liver through regulation of the PI3K/AKT pathway, leading to progression of hepatocellular carcinoma (HCC) [28,29]. In contrast, MAO-A suppresses HCC metastasis through EGFR signaling [30]. Norepinephrine increases hepatic glycogenolysis and gluconeogenesis [31] and up-regulates mitogenic signaling pathways, such as PI3K/AKT and MAPK/ERK [32], promoting cell proliferation [33,34]. Serotonin also promotes proliferation in hepatoma cells through AKT phosphorylation and inhibits autophagy [35,36].

Little is known about the association of the monoamine biosynthetic and metabolic pathway with viral infections. In addition to our previous reports on the association of DDC with HCV [7] and SARS-CoV-2 [37] infections, there is a study supporting increases of MAO-B transcription upon Simian immunodeficiency virus infection in macaque brain [38]. Moreover, Rotavirus (RV) and Dengue virus (DENV) have been shown to stimulate 5-HT release from the host cells [39,40]. Similarly, Enterovirus 71 (EV71) infection resulted in elevated plasma levels of NE and EN in patients with neurological complications and the two catecholamines enhanced virus titers and infectivity [41]. On the other hand, infection of newborn mice with Coxsackie type B4 virus or yellow fever virus disrupted catecholamine biosynthesis in the brain [42]. In turn, viral entry is known to be facilitated by dopamine receptors D2 or D4 in the case of DENV [43,44] and by 5-HT receptors in the case of HCV [45], Reovirus [46] and JC Polyomavirus [47], while the lytic switch of Epstein-Barr Virus is regulated by 5-HT receptors [48].

Taking into account the emerging role of the catecholamines and indolamine (serotonin) biosynthetic/metabolic pathway in viral infections and pathogenesis, in the present study, we aimed to investigate the relationship of HCV with different steps of this pathway in human hepatoma cells. Besides the previously shown association between DDC-PI3K interaction and HCV replication in hepatocytes, here we set out to elucidate the potential impact of the biosynthetic role of DDC along with other catecholamine and serotonin pathway-related proteins on virus proliferation. Henceforth, by either silencing or chemical inhibition, we regulated the activity of the pathway-associated proteins, including biosynthetic enzymes and transporters, and furthermore, we exogenously applied their substrates as well as their products. In addition, we further characterized the underlying mechanisms of the previously reported DDC regulation by HCV and examined the impact of viral infection on mRNA and protein expression of the other enzymes of the biogenic amine metabolic pathway.

## 2. Materials and Methods

### 2.1. Cell Culture

Huh7.5 [49] and Huh7-Lunet [50] cells, as well as Huh5-2 stable cell line harboring the subgenomic reporter replicon of genotype 1b (Con1) [51], were cultured in high glucose (25 mM) Dulbecco’s modified minimal essential medium (Thermo Fisher Scientific, Waltham, MA, USA), supplemented with 0.1 mM non-essential amino acids, 2 mM l-glutamine, 100 U/mL penicillin, 100 μg/mL streptomycin, and 10% (*v/v*) heat inactivated fetal calf serum (referred to as complete DMEM). For Huh5-2 cells, complete DMEM was supplemented with G418 at 500 μg/mL. Cells were cultured at 37 °C in a 5% (*v/v*) CO_2_ environment.

### 2.2. Viruses and Plasmid Constructs

Plasmids pFK-Jc1 and pFK-i389RLuc2ACore-30-Jc1 (JcR2a), carrying the full-length chimeric HCV genotype 2a genome Jc1 (J6/CF codons 1 to 846 combined with JFH1 codons 847 to 3033) without or with a Renilla luciferase (RLuc) reporter, respectively, have been described previously [52,53]. For *DDC* silencing, the psi-LVRH1GP/shDDC (shDDC) plasmid, expressing a short hairpin RNA (shRNA) targeting *DDC* mRNA (5′-GCTCCTTTGACAATCTCTTAG-3′), or the control vector (shControl), expressing a scramble negative-control shRNA (5′-GCTTCGCGCCGTAGTCTTA-3′), were obtained from GeneCopoeia. The plasmid vectors for mammalian expression of the HCV NS4B and NS5A protein, pEGFP-NS4B and pcDNA3-NS5A, respectively, were previously described [54,55].

### 2.3. In Vitro Transcription

Ten micrograms of viral constructs were linearized with MluI and were used for in vitro transcription as described previously [56]. In brief, plasmid DNA was extracted with phenol and chloroform, precipitated with ethanol and dissolved in RNase-free water. In vitro transcription was performed in 80 mM HEPES (pH 7.5) containing 12 mM MgCl2, 2 mM spermidine, 40 mM dithiothreitol (DTT), 3.125 mM of each nucleoside triphosphate, 1 U/μL of RNasin (Promega Corporation, Madison, WI, USA), 0.1 μg plasmid DNA/μL, and 0.6 U/μL of T7 RNA polymerase (Promega). Afterwards, 1.2 U of RNase-free DNase (Promega) per μg of plasmid DNA was added to terminate transcription. The viral RNA was extracted with acidic phenol and chloroform, precipitated with isopropanol, and dissolved in RNase-free water.

### 2.4. Transfection Assays

For viral protein overexpression, Huh7-Lunet cells were electroporated with 60 μg of plasmids pEGFP-NS4B, pcDNA3-NS5A, or the empty vector pcDNA3 (In vitro gen). In *DDC* silencing experiments, Huh7.5 cells were electroporated with 60 μg of the shDDC or shControl expressing plasmids. 4 × 10^6^ cells were detached with trypsin, washed with PBS 1×, resuspended in Cytomix [57] containing 2 mM ATP and 5 mM glutathione, mixed with plasmid DNA and electroporated at 220 V, 975 μF with a Gene Pulser system (Bio-Rad, Hercules, CA, USA). The cells were immediately transferred to 20 mL of complete DMEM and subsequently seeded as required for the assay.

### 2.5. Preparation and Titration of Virus Stocks and Infection Assays

HCV virus stocks were generated in Huh7-Lunet cells as described elsewhere [58]. In brief, virus stocks were generated by electroporation of in vitro transcribed viral RNAs, and the supernatants of transfected cells were harvested 24, 48, 72, and 96 h post-transfection (p.t). After filtration of the supernatants through 0.45-μm-pore-size filters, the virus particles were concentrated by ultracentrifugation through a self-generating iodixanol gradient. The collected fractions containing virus particles were further concentrated using Centricon Plus-70 centrifugal filter devices (Merck-Millipore, Burlington, MA, USA) according to the manufacturer’s instructions. HCV was titrated as described elsewhere [59], using the JFH1 NS5A-specific mouse monoclonal antibody 9E10 [59]. Infectivity titers were expressed as the 50% tissue culture infective dose (TCID50)/mL. Virus stocks were used to infect naive Huh7.5 cells. The culture medium was exchanged 4 h post-virus inoculation.

### 2.6. Gel Electrophoresis and Western Blot Analysis

Denaturing SDS-polyacrylamide gel electrophoresis and Western blotting was performed as described elsewhere [60]. Dilutions of 1:1000 for HCV NS5A monoclonal antibody (9E10) [59], 1:200 for HCV NS4B rabbit polyclonal antibody (NS4B-N, raised against the 55 N-terminal amino acids of NS4B, previously described in [54]), 1:1000 for DDC rabbit polyclonal antibody for the detection of the ~120 kDa DDC immunoreactive SDS-resistant protein (anti-DDC C-T, raised against the 22 C-terminal amino acids of human DDC, previously described in [11], 1:1000 for DDC mouse monoclonal antibody for the detection of the ~50 kDa DDC monomer (clone 8E8; Santa Cruz Biotechnology, Heidelberg, Germany), 1:500 for TH mouse monoclonal antibody (clone F-11; Santa Cruz Biotechnology), 1:200 for DBH mouse monoclonal antibody (clone A-9; Santa Cruz Biotechnology), 1:1000 for VMAT2 mouse monoclonal antibody (clone H-12; Santa Cruz Biotechnology), 1:500 for MAO-B mouse monoclonal antibody (clone D-6; Santa Cruz Biotechnology), and 1:6000 for pan-actin mouse monoclonal antibody (Merck-Millipore, Burlington, MA, USA), respectively, were used. A dilution of 1:2000 for secondary anti-mouse or anti-rabbit horseradish peroxidase-conjugated antibodies (Cell Signaling, Leiden, The Netherlands) was used.

### 2.7. Luciferase Assay

Renilla (R-Luc) and Firefly (F-Luc) luciferase activities were determined in cell lysates, using 12 μΜ coelenterazine in an assay buffer (50 mM potassium phosphate, pH 7.4, 500 mM NaCl, and 1 mM EDTA) or Luciferase Assay system (Promega Corporation, Madison, WI, USA), respectively, and measurements were taken in a GloMax 20/20 single-tube luminometer (Promega Corporation, Madison, WI, USA) for 10 s, as previously described [7]. Luciferase activity was normalized to the total protein amount quantified with the Bradford assay reagent (Bio-Rad, Hercules, CA, USA).

### 2.8. Measurement of Intracellular ATP Levels

Intracellular ATP was measured using the ViaLight HS BioAssay kit (Lonza, Basel, Switzerland) according to the manufacturer’s protocol, in a GloMax 20/20 single-tube luminometer (Promega Corporation, Madison, WI, USA) for 1 s, as recommended by the manufacturer. ATP levels were normalized to total protein amounts.

### 2.9. RNA Quantification by Reverse Transcription-Quantitative PCR (RT-qPCR)

Total RNA extraction from cells was performed using nucleoZOL reagent (Macherey-Nagel, Duren, Germany) according to the manufacturer’s instructions. cDNA synthesis was carried out with Moloney murine leukemia virus reverse transcriptase (Promega Corporation, Madison, WI, USA) based on the manufacturer’s protocol. For HCV positive-strand RNA quantitation, reverse transcription (RT) reactions included the HCV-specific primer JFH1-354R, as well as the primer YWHAZ-R (Table 1), specific for the housekeeping gene 14-3-3-zeta polypeptide (YWHAZ) used as internal control (3.5 pmol/μL of each primer). For the quantification of cellular transcripts, oligo(dT) primers (New England Biolabs, Ipswich, MA, USA) were included. Real-time quantitative PCR was performed using Luna^®^ Universal qPCR Master Mix (New England Biolabs, Inc. Ipswich, MA, USA), as well as primer pairs specific for the HCV IRES (JFH1-276F and JFH1-354R), the exons 10-12 of full-length *DDC* mRNAs, *TH*, *DBH*, *MAO-A*, *MAO-B*, *VMAT2*, *OCT1*, *NRF2*, *HO-1* and *VEGFA* mRNAs. The *YWHAZ* housekeeping gene was used as a normalization control in all qPCR reactions, as its expression was not affected upon viral infection.

### 2.10. Indirect Immunofluorescence

Indirect immunofluorescence analysis of human DDC and HCV NS5A in Huh7-Lunet cells was performed as previously described [7]. Cells were seeded onto glass coverslips in 24-well plates (5 × 10^4^ cells/well). At 48 h post-electroporation with plasmid DNA, cells were fixed with 3% paraformaldehyde for 10 min at room temperature and permeabilized by incubation in PBS supplemented with 0.5% Triton X-100 for 5 min. Staining of DDC was performed by using an anti-DDC C-T polyclonal antibody at a dilution of 1:20, while staining of HCV NS5A was performed by using the mouse monoclonal antibody 9E10 [59] at a dilution of 1:1000. Bound primary antibodies were detected by using goat anti-mouse antibodies conjugated to Alexa-Fluor 488, or goat anti-rabbit antibodies conjugated to Alexa-Fluor 546 at a dilution of 1:1000. DNA was stained with Hoechst 33258 (Thermo Fisher Scientific). HCV NS4B was detected through GFP fluorescence. Images were acquired with the Leica TCS-SP8 confocal microscope. Fluorescence quantitation and colocalization analysis was carried out using Icy software [61,62]. Pearson’s correlation coefficient and Manders’ colocalization coefficients were calculated using Colocalization Studio plugin.

### 2.11. ELISA Assay

Dopamine was quantified in cell supernatants using the Dopamine ELISA kit (IBL International, Hamburg, Germany), according to the manufacturer’s instructions. To the supernatants collected after treatment, 30 μM EDTA and 110 μM L-ascorbic acid were added to prevent dopamine oxidation, and subsequently samples were centrifuged at 2000 *g* for 20 min at 4 °C for removal of cell debris.

### 2.12. Chemicals

L-Dopa, 5-Hydroxytryptophan (5-HTP), dopamine (DA), serotonin (5-HT), clorgyline, phenelzine, reserpine, forskolin, phorbol 12-myristate 13-acetate (PMA), L-ascorbic acid and L-Glutathione reduced were obtained from Sigma-Aldrich (Saint Louis, MO, USA). Nepicastat, norepinephrine (NE) and prochlorperazine (PCZ) were obtained from Cayman Chemical (Ann Arbor, MI, USA).

### 2.13. Statistical Analysis

In all diagrams, bars denote mean values of at least 3 independent experiments in triplicate and error bars standard deviation. Statistical analyses were performed using Student’s t-test and results with *p* < 0.05 were considered as statistically significant. Calculations were carried out using Excel Microsoft Office^®^ (Microsoft Corporation, Redmond, WA, USA) or Prism (GraphPad Software, Inc., San Diego, CA, USA).

### 2.14. Ethics Statement

This material is the authors’ own original work, which has not been published in whole or in part elsewhere. All authors have been personally and actively involved in substantive work leading to the manuscript, and will hold themselves responsible for its content. This study does not involve humans or animals.

## 3. Results

### 3.1. Silencing of L-Dopa Decarboxylase (DDC) Increases HCV Replication and Suppresses the Antiviral Effect of DDC Substrates

**Figure 1:**
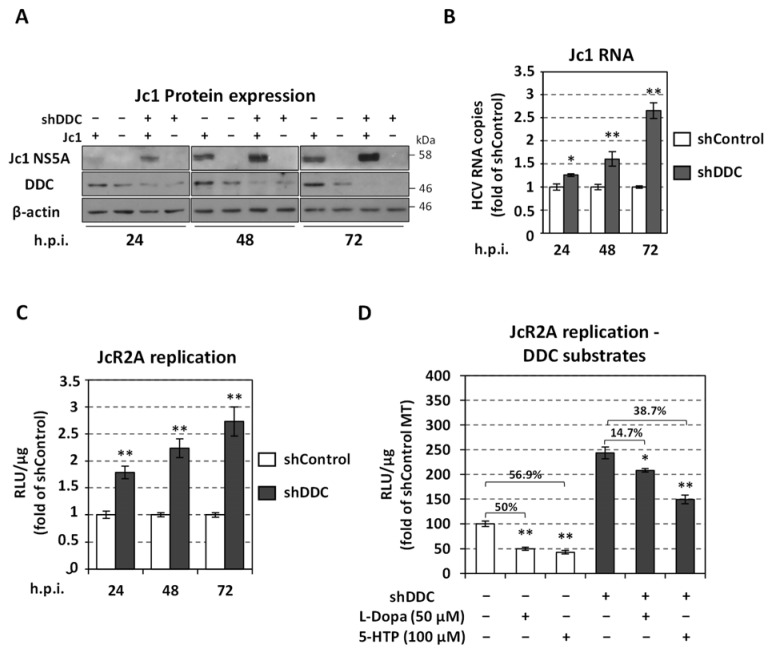
Effect of *DDC* silencing on HCV replication and on the antiviral activity of DDC substrates. Huh7.5 cells were electroporated with psi-LVRH1GP/sh-DDC (shDDC) plasmid or the control vector (shControl), inoculated 24 h p.t. with Jc1 (MOI = 1) (**A**,**B**) or the reporter virus JcR2A (MOI = 0.5) (**C**,**D**) for 4 h, and lysed at the indicated post-infection hours (h.p.i). (**A**) Western blot analysis of shDDC (+) and shControl (−) cells, using anti-DDC antibody, anti-NS5A antibody for the detection of Jc1 infection and β-actin as loading control. Mock-infected cells were used in parallel and are symbolized as Jc1 (−). A representative experiment of three independent triplicates is shown. (**B**) Jc1 positive-strand RNA levels were quantified by RT-qPCR and YWHAZ mRNA was used for normalization. Values from shControl cells were set to one at each time-point. (**C**) JcR2A replication-derived Renilla luciferase (R-Luc) activity was quantified by chemiluminescence-based assay and expressed as relative light units (RLU) per μg of total protein amount. Values from shControl cells were set to one at each time-point. (**D**) JcR2A replication-derived R-Luc activity was quantified in shDDC (+) and shControl (−) cells, which were treated with 50 μM L-Dopa or 100 μM 5-HTP for 72 h starting from 4 h post-virus inoculation or were mock-treated (MT). Values from shControl MT cells were set to one. Percentages of viral-replication reduction after treatment, are shown above brackets. Bars represent mean values from three independent experiments in triplicate. Error bars indicate standard deviations. * *p* < 0.01, ** *p* < 0.001 vs. Control.

Having previously identified DDC as a novel cellular factor regulated by HCV infection and that in turn DDC overexpression/chemical inhibition affects viral RNA replication [7], hereby we performed gene silencing to confirm the negative effect of DDC on HCV replication and to examine whether this effect is mediated by the biosynthetic role of DDC. To silence DDC, human hepatoma Huh7.5 cells were electroporated with an shDDC plasmid vector or a scramble negative control shRNA plasmid (shControl) and subsequently infected with Jc1 or the reporter JcR2A virus. The silencing of *DDC* was shown to positively affect HCV proliferation with a significant increase up to ~3-fold observed at the level of Jc1 NS5A protein (Figure 1A), viral positive-strand RNA (Figure 1B) and JcR2A replication-derived Renilla luciferase (R-Luc) activity (Figure 1C) in cell lysates (see also Figure S1A,B; replication kinetics of the viruses). *DDC* silencing was confirmed by measuring intracellular DDC mRNA (Figure S1C) and protein levels (Figure 1A). Next, we confirmed that the favorable role of *DDC* silencing on HCV is exerted at the level of viral RNA replication by introducing the subgenomic JFH1 replicon in stable transfected cell lines expressing shDDC or shControl RNA (Figure S1D,E).

Moreover, *DDC* silencing attenuates the negative impact of DDC enzymatic substrates on JcR2A replication (Figure 1D). Specifically, non-cytotoxic concentrations of L-Dopa and 5-HTP (Figure S2A,B) reduced virus-derived R-Luc activity in shControl-electroporated cells by 50% and 57%, respectively (Figure 1D), in consistence with our previous data [7]. This reduction was confirmed for Jc1 RNA and protein (Figure S2C,D). However, a significantly lower effect was observed for L-Dopa (15%) and 5-HTP (39%) (Figure 1D) in shDDC-electroporated cells. This suggests that DDC affects HCV replication at least through the processing of L-Dopa and 5-HTP.

### 3.2. Exogenous Application of the DDC Protein Products Dopamine and Serotonin Decreases HCV Replication

**Figure 2:**
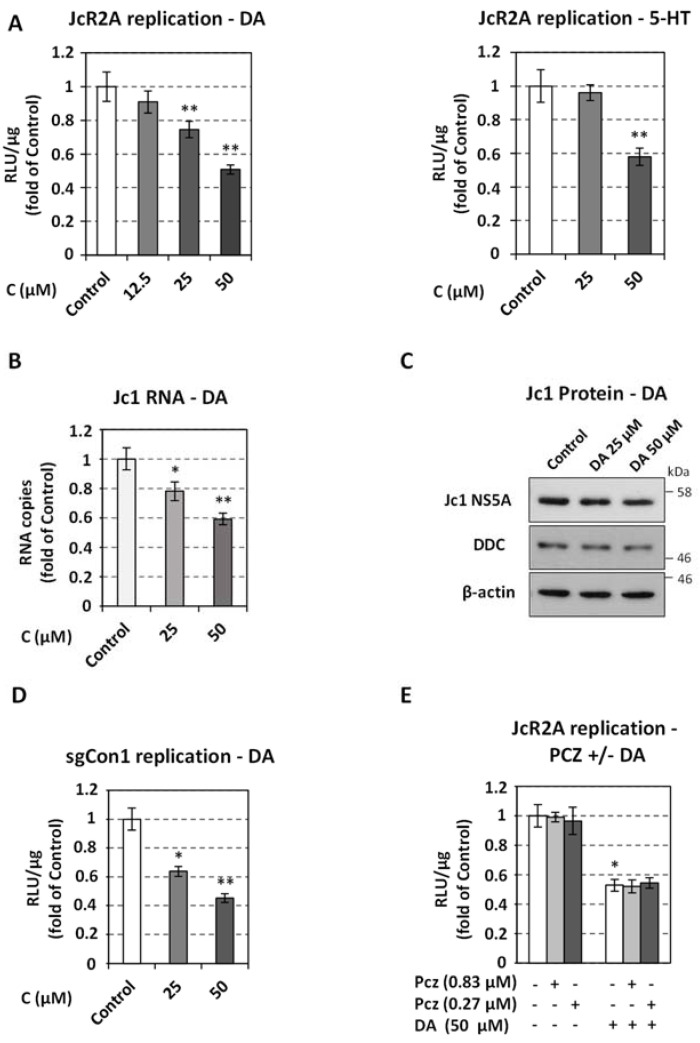
Treatment of HCV-infected cells with DDC products. Huh7.5 cells were inoculated with JcR2A (MOI = 0.5) (**A**,**E**) or Jc1 (MOI = 1) (**B**,**C**) for 4 h, and then treated for 72 h, with the indicated concentrations of DA, 5-HT, PCZ or mock-treated (Control). (**A**,**E**) Virus replication-derived R-Luc activity was determined in cells cultured in the presence or absence of DA (left) or 5-HT (right) and expressed as RLU/μg of total protein amount. (**B**) Jc1 positive-strand RNA levels, in cells treated with DA or mock-treated, were quantified by RT-qPCR and YWHAZ mRNA was used for normalization. (**A**,**B**) Values from control cells were set to one. Bars represent mean values from three independent experiments in triplicate. Error bars indicate standard deviations. * *p* < 0.01, ** *p* < 0.001 vs. Control. (**C**) Western blot analysis was performed in lysates of Jc1-infected cells treated for 72 h or mock-treated (Control) with 25 μM or 50 μM of DA, using anti-HCV NS5A, anti-DDC, or anti-β-actin antibodies. β-actin was used as loading control. A representative experiment of three independent repetitions is shown. (**D**) Effect of DA on HCV genotype 1b replication. Huh5-2 cells, harboring HCV genotype 1b (Con1) subgenomic replicon, were treated for 72 h with the indicated concentrations of DA, or were mock-treated (Control). Viral RNA replication-derived firefly luciferase activity was determined and expressed as RLU/μg of total protein. Values from control cells were set to one. (**E**) Combinatory effect of DA and PCZ on JcR2A replication. Values from infected mock-treated cells were set to one. (**D**,**E**) Bars represent mean values from three independent experiments in triplicate. Error bars indicate standard deviations. * *p* < 0.01, ** *p* < 0.001 vs. Control.

We then examined whether the virus is affected by the exogenous addition of DDC products dopamine (DA) and serotonin (5-HT). Treatment of JcR2A-infected Huh7.5 cells with DA or 5-HT for 72 h starting from 4 h post-virus inoculation, reduced virus-derived R-Luc activity by ~2-fold (Figure 2A, see also Figure S3A,B for cytotoxicity profile). Accordingly, lower viral RNA (Figure 2B) and protein (Figure 2C) levels were detected in DA-treated cells as compared to mock-treated (Control) ones. The negative effect of DA on viral RNA replication was confirmed in the HCV genotype 1b (Con1) subgenomic replicon cell line (Figure 2D). DA did not significantly alter DDC mRNA and protein levels in both infected and mock-infected cells (Figure 2C and Figure S4A). To discriminate if DA negatively influences HCV through the dopaminergic receptor signaling, we applied prochlorperazine (PCZ), an antagonist of the D2 dopamine receptor, to JcR2A-infected cells. We selected to study D2 as it has been detected and well-characterized in the liver [63,64,65]. PCZ had no impact on viral replication either in the absence or presence of exogenous dopamine (Figure 2E). The above data combined suggest that the effect of dopamine on HCV is possibly exerted by its intracellular production and uptake and not through dopamine receptor signaling.

### 3.3. HCV Replication Is Enhanced by the Use of an Inhibitor of the Monoamine Transporter VMAT2

**Figure 3:**
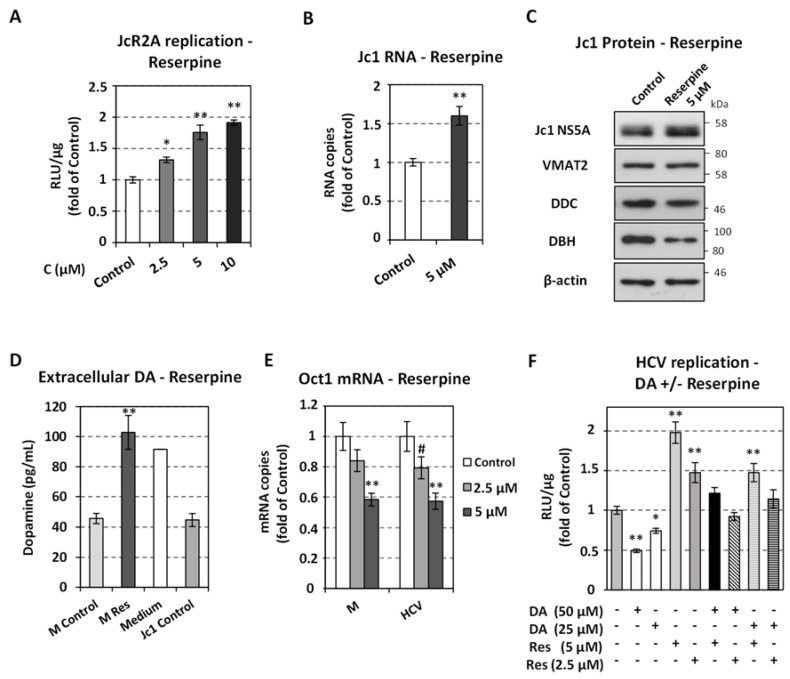
Effect of Reserpine on HCV replication. Huh7.5 cells were inoculated with JcR2A (MOI = 0.5) (**A**,**F**) or Jc1 (MOI = 1) (**B**–**E**) for 4 h, or mock infected (M) and subsequently treated for 72 h with the indicated concentrations of reserpine, or mock treated (Control). (**A**) Virus replication-derived R-Luc activity was determined in cell lysates and expressed as RLU/μg of total protein amount. (**B**) Jc1 positive-strand RNA levels were quantified by RT-qPCR and YWHAZ mRNA was used for normalization. (**A**,**B**) Values from infected mock-treated cells were set to one. Bars represent mean values from three independent experiments in triplicate. Error bars indicate standard deviations. # *p* < 0.05, * *p* <0.01, ** *p* < 0.001 vs. Control. (**C**) Western blot analysis was performed in lysates of Jc1-infected cells treated for 72 h or not (Control) with 5 μM reserpine, using anti-HCV NS5A, anti-VMAT2, anti-DDC, anti-DBH or anti-β-actin antibodies. β-actin was used as loading control. A representative experiment of three independent repetitions is shown. (**D**) Reserpine inhibits dopamine uptake. Huh7.5 cells were inoculated with Jc1 (MOI = 1) for 4 h, or mock infected (M), and were further cultured for 72 h with 5 μΜ reserpine or the solvent (mock-treated cells, Control), and their supernatants were collected. Extracellular Dopamine was quantified by non-competitive enzyme immunoassay and compared to the counterpart of the cell culture medium (Medium). (**E**) Oct1 mRNA levels were determined by RT-qPCR and normalized to YWHAZ mRNA. Values from control cells were set to one. (**F**) Combinatory effect of reserpine and DA on JcR2A replication. Virus replication-derived R-Luc activity was determined in cell lysates and expressed as RLU/μg of total protein amount. Values from non-treated cells (Control) were set to one. (**D**–**F**) Bars represent mean values from three independent experiments in triplicate. Error bars indicate standard deviations. # *p* < 0.05, * *p* < 0.01, ** *p* < 0.001 vs. Control.

To directly address the importance of catecholamines and serotonin uptake and storage on HCV replication, we inhibited the activity of VMAT2. VMAT2 is responsible for the uptake of catecholamines and serotonin from extracellular medium through up-regulation of their transporters, and for their transfer in storage vesicles, where DA is converted to NE by the enzyme DBH [66,67,68,69]. Specifically, Huh7.5 cells mock-infected or infected with the reporter JcR2A or the Jc1 virus were subsequently treated with non-cytotoxic concentrations of the VMAT-specific irreversible inhibitor reserpine (Figure S3D) [70,71]. Reserpine has been shown to deplete intracellular catecholamine stores [70,71] by inhibiting dopamine and norepinephrine transporter expression [72,73] and activity [69] and thus the uptake of catecholamines, through a VMAT- and VMAT-containing catecholamine storage vesicles-dependent mechanism [66,67,68,69]. In agreement with the above, reserpine reduced the levels of DBH protein in Huh7.5 cells (Figure 3C), possibly due to the inhibition of dopamine import in the cytoplasmic vesicles.

Reserpine enhanced the replication of HCV as the JcR2A replication-derived R-Luc activity was increased by up to 2-fold (Figure 3A). Similarly, Jc1 RNA and protein levels were higher in reserpine-treated cells, as compared to mock-treated (Control) cells (Figure 3B,C). A comparable up-regulation was observed in the subgenomic replicon system (Figure S5A). VMAT2 protein expression was confirmed in Huh7.5 cells (Figure 3C), in agreement to its previously reported detection in hepatocytes. These data probably suggest that VMAT2 negatively correlates with HCV replication. Moreover, in reserpine-treated cells the extracellular dopamine levels were 2.25-fold higher than in control (mock-treated, Control) cells, as determined by ELISA assay in cell supernatants (Figure 3D), and similar to the dopamine levels in a plain culture medium not incubated with cells (Medium). Thus, cells appeared to uptake/metabolize the medium-containing dopamine in agreement with previous studies [74], while reserpine seemed to completely abrogate this process. On the other hand, HCV infection did not cause any significant change in the extracellular dopamine levels (Figure 3D, Jc1 Control). To further address the effect of reserpine on the uptake of dopamine by the cell, we examined the expression of *OCT1*, a major catecholamine transporter in hepatocytes [25,26]. Indeed, reserpine reduced OCT1 mRNA levels, as determined by qPCR in the cell lysates of both infected and mock-infected Huh7.5 cells (Figure 3E). The above data combined suggest that reserpine may increase viral replication through a reduction of OCT1 transporters expression that lowers the intracellular levels of catecholamines. This premise is substantiated by the finding that reserpine abolished the negative effect of exogenously supplied dopamine on viral replication (Figure 3F). Finally, a reserpine-mediated HCV-replication increase occurred concomitantly with a reduction in DDC mRNA levels (Figure S4B), which was expected based on the negative correlation between viral replication and *DDC* expression reported in our previous [7] and current studies. In mock-infected cells, *DDC* expression was not altered by reserpine.

### 3.4. The Role of DBH, the Enzyme Catalyzing the Conversion of Dopamine to Norepinephrine, for HCV Replication

**Figure 4:**
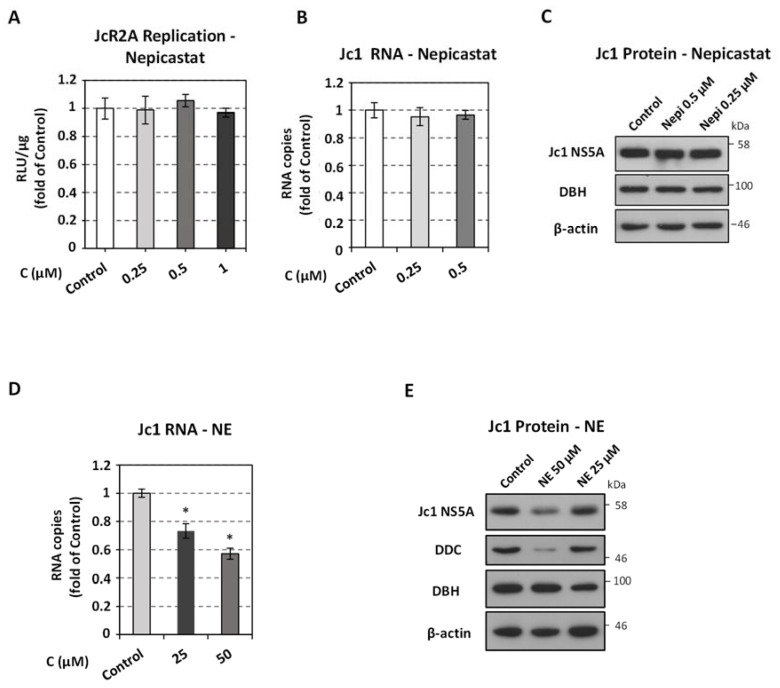
Effect of DBH inhibitor nepicastat and DBH product NE on HCV replication. (**A**–**C**) Huh7.5 cells were inoculated with JcR2A (MOI = 0.5) (**A**) or Jc1 (MOI = 1) (**B**–**E**) for 4 h and subsequently treated or not (Control) for 72 h with different concentrations of nepicastat (**A**–**C**) or NE (**D**,**E**), or were mock treated (Control). (**A**) Virus replication-derived R-Luc activity was determined in cells cultured in the presence or absence of nepicastat and expressed as RLU/μg of total protein amount. (**B**,**D**) Jc1 positive-strand RNA levels, in cells treated with nepicastat (**B**) or NE (**D**), or mock-treated, were quantified by RT-qPCR and YWHAZ mRNA was used for normalization. (**A**,**B**,**D**) Values from infected non-treated cells were set to one. Bars represent mean values from three independent experiments in triplicate. Error bars indicate standard deviations. * *p* < 0.01 vs. Control. (**C**,**E**) Western blot analysis was performed in lysates of Jc1-infected cells treated for 72 h with nepicastat (**C**), or NE (**E**) or mock-treated (Control) using anti-HCV NS5A, anti-DBH, anti-DDC or anti-β-actin antibodies. β-actin was used as loading control. A representative experiment of three independent repetitions is shown.

Treatment of HCV-infected cells with non-cytotoxic concentrations of the DBH-specific inhibitor nepicastat (Figure S3E), which inhibits the biosynthesis of norepinephrine (NE) [75], did not seem to affect either HCV replication-derived luciferase activity or viral RNA and protein levels (Figure 4A–C and Figure S5B). On the other hand, externally provided NE reduced viral replication in infected cells (Figure 4D,E), despite that NE-treatment enhanced the intracellular ATP content (Figure S3F), possibly through up-regulation of aerobic glycolysis [31,76]. Moreover, treatment with reserpine that has been previously reported to inhibit the uptake of NE [69,72] alleviated the negative effect of NE on viral replication (Figure S5C), similarly to our observation for dopamine and reserpine co-treatment (Figure 3F). Finally, NE reduced DDC mRNA levels (Figure S4C), which was also reverted by the co-treatment with reserpine (Figure S4D). The above results suggest that when NE is restricted inside the cytosolic vesicles, it cannot influence HCV replication, whereas its uptake and accumulation in the cell cytosol leads to the inhibition of viral replication. 

### 3.5. Inhibition of MAO Monoamine Degradation Enzymes Reduces HCV Replication

**Figure 5:**
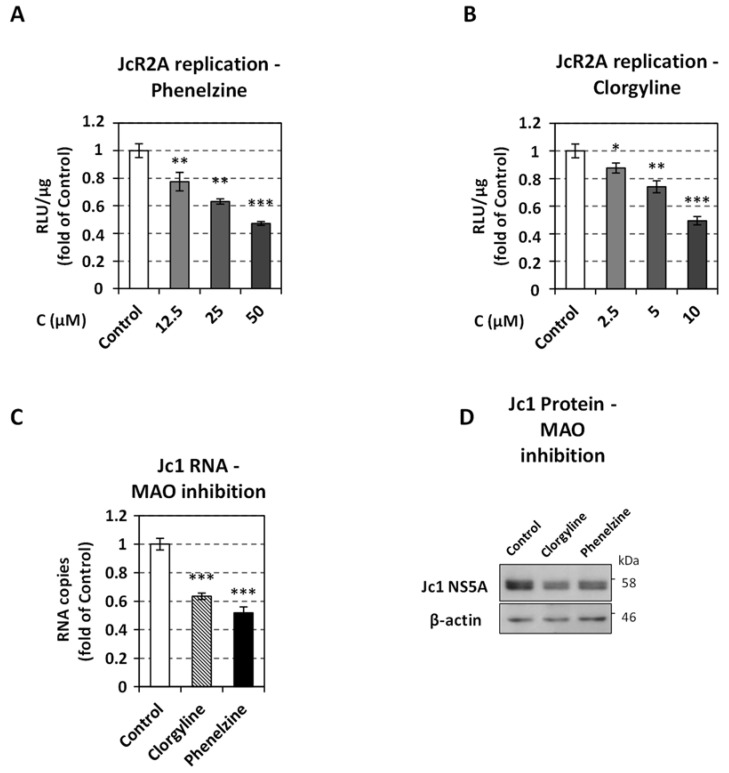
Effect of MAO inhibition on HCV replication. (**A**,**B**) Huh7.5 cells were inoculated with JcR2A (MOI = 0.5) for 4 h and subsequently treated for 72 h with the indicated concentrations of phenelzine (**A**) or clorgyline (**B**), or mock-treated (Control). Virus replication-derived R-Luc activity was determined in cell lysates and expressed as RLU/μg of total protein amount. (**C**) Huh7.5 cells were inoculated with Jc1 (MOI = 1) for 4 h and subsequently treated for 72 h with 10 μΜ clorgyline, 50 μM phenelzine, or mock-treated (Control). Viral positive-strand RNA levels were quantified by RT-qPCR and YWHAZ mRNA was used for normalization. (**A**–**C**) Values from infected non-treated cells were set to one. Bars represent mean values from three independent experiments in triplicate. Error bars indicate standard deviations. * *p* < 0.05, ** *p* < 0.01, *** *p* < 0.001 vs. Control. (**D**) Western blot analysis of HCV NS5A was performed in lysates of the Jc1-infected cells treated for 72 h with 10 μΜ clorgyline, 50 μM phenelzine, or mock-treated (Control). β-actin was used as loading control. A representative experiment of three independent repetitions is shown.

Thus far, our data suggested that the accumulation of catecholamines in the cell cytosol negatively affected HCV replication. Part of the accumulated cytosolic-located catecholamines can either undergo auto-oxidation, producing toxic quinones that can also generate ROS [77], or can be deaminated by MAO enzymes. To examine if MAO function could contribute to the regulation of HCV replication, we performed an inhibition of MAO-A and MAO-B in Huh7.5 cells. MAO-A has a higher affinity to 5-HT, while both MAO isoforms metabolize catecholamines [20] and their inhibition causes the accumulation of these monoamines in the cytoplasm. We observed that virus replication was up to 2-fold lower in the presence of the MAO-A and MAO-B irreversible inhibitor phenelzine (Figure 5A and Figure S3G) or the MAO-A irreversible selective inhibitor clorgyline (Figure 5B and Figure S3G), compared to control-treated cells. The impact of MAO inhibition on HCV replication was confirmed in Jc1-infected cells by quantifying viral RNA and protein levels (Figure 5C,D), suggesting that MAO activity alleviates the inhibitory effect of catecholamines on HCV replication. These results, combined with data showing the DDC-mediated inhibition of HCV by L-Dopa and 5-HTP, suggest that the antiviral effect in hepatocytes is mediated at least by the accumulation of the endogenous produced and imported catecholamines.

### 3.6. Induction of the Catecholamine and Serotonin Biosynthetic/Metabolic Pathway Down-Regulates HCV Replication

**Figure 6:**
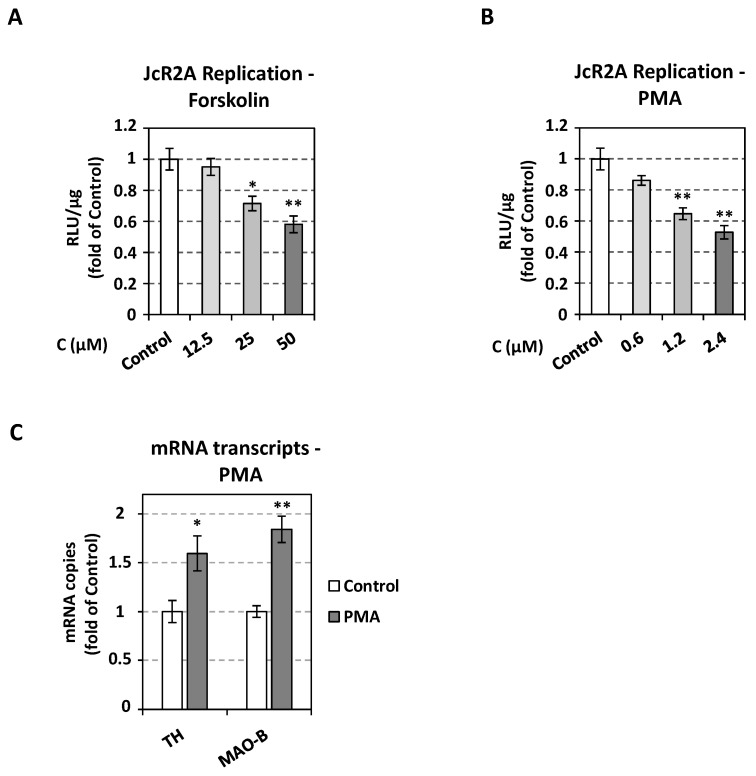
Effect of PKA and PKC activation on HCV replication. (**A**,**B**) Huh7.5 cells were inoculated with JcR2A (MOI = 0.5) for 4 h and then treated for 72 h with the indicated concentrations of Forskolin (**A**), PMA (**B**), or mock-treated (Control). Virus replication-derived R-Luc activity was determined and expressed as RLU/μg of total protein amount. (**C**) Effect of PMA treatment on TH and MAO-B transcription. Huh7.5 cells were treated with 2.4 μΜ PMA or mock-treated (Control). TH and MAO-B mRNA levels were quantified by RT-qPCR and normalized to the mRNA levels of the housekeeping gene YWHAZ. In all panels, values from infected non-treated cells were set to one. Bars represent mean values from three independent experiments in triplicate. Error bars indicate standard deviations. * *p* < 0.01, ** *p* < 0.001 vs. Control.

To further confirm the negative implication of the catecholamine producing enzymes on HCV, we applied inducers of protein kinase A (PKA) and protein kinase C (PKC). The activation of these proteins enhances the accumulation of catecholamines through phosphorylation of DDC [78], stimulation of TH transcription and enzymatic activity [12] or increasing MAO-B gene expression and activity [79]. Treatment of JcR2A-infected Huh7.5 cells for 72 h with different concentrations of PKA activator forskolin or PKC activator phorbol 12-myristate 13-acetate (PMA) resulted in up to a 2-fold reduction of virus-derived R-Luc activity (Figure 6A,B and Figure S3H). A PMA-dependent TH and MAO-B mRNA upregulation was observed in Huh7.5 cells (Figure 6C), confirming the effect of the protein kinase inducer on the catecholamine biosynthetic/metabolic pathway under our experimental conditions.

### 3.7. Association of Catecholamine-Mediated HCV Regulation with Cellular Redox Homeostasis

Catecholamines in the cytoplasm are autoxidized, producing damaging quinones that also generate ROS. Auto-oxidation can be limited by MAO that oxidatively deaminate catecholamines and serotonin, generating H_2_O_2_ [12,13,80]. Concerning HCV, there are reports showing an inhibitory effect of elevated ROS levels on HCV replication [81,82], as well as the sensitivity of viral core and NS5A proteins to oxidative stress-induced degradation [83].

**Figure 7:**
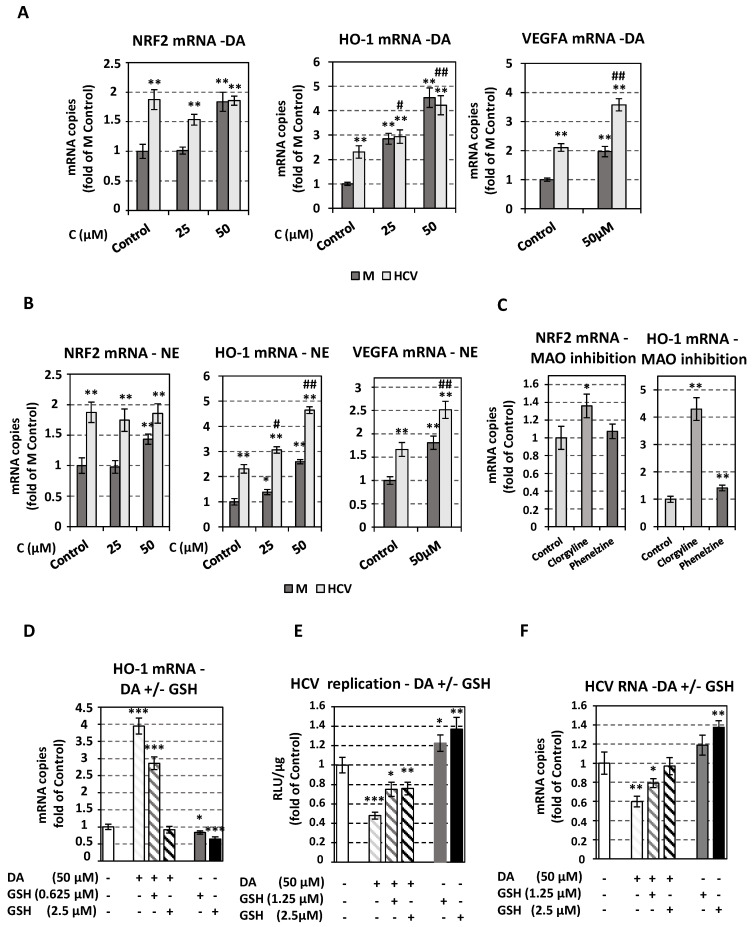
Effect of oxidative stress related to catecholamine biosynthesis and metabolism on HCV replication. Huh7.5 cells were inoculated with Jc1 (MOI = 1) (**A**,**B**,**D**,**F**) or JcR2A (MOI = 0.5) (**E**) for 4 h or mock-infected (M) and subsequently treated or not (Control) for 72 h with different concentrations of catecholamines (DA, NE), MAO inhibitors (clorgyline, phenelzine), or GSH. (**A**,**B**) NRF2, HO-1, VEGFA mRNA levels in infected or mock-infected cells, treated with DA (**A**) or NE (**B**), were quantified by RT-qPCR and normalized to the mRNA levels of the housekeeping gene YWHAZ. Values from M Control cells were set to one. (**C**) NRF2 and HO-1 mRNA levels treated with clorgyline or phenelzine mock-infected cells, were quantified by RT-qPCR and YWHAZ mRNA was used for normalization. Values from Control cells were set to one. Bars represent mean values from at least three independent experiments in triplicate. Error bars indicate standard deviations. * *p* < 0.01, ** *p* < 0.001 vs. M (mock-infected) Control, # *p* < 0.01, ## *p* < 0.001 vs. HCV-infected Control. (**D**–**F**) Effect of DA and GSH combinatory treatment on the expression of HO-1 antioxidant gene and on HCV replication, in infected cells. (**D**) HO-1 mRNA levels were quantified by RT-qPCR and YWHAZ mRNA was used for normalization. (**E**) Virus replication-derived R-Luc activity was determined in cell lysates and expressed as RLU/μg of total protein amount. (**F**) Jc1 positive-strand RNA levels were quantified by RT-qPCR and YWHAZ mRNA was used for normalization. In panels D–F, values from infected non-treated cells were set to one. Bars represent mean values from at least three independent experiments in triplicate. Error bars indicate standard deviations. * *p* < 0.05, ** *p* < 0.01, *** *p* < 0.001 vs. Control.

Therefore, we sought to investigate whether the suppression of HCV replication by catecholamine biosynthesis is mediated by ROS production. First, we confirmed that the oxidative stress is induced in Huh7.5 cells treated with dopamine, norepinephrine or MAO inhibitors clorgyline and phenelzine, as detected by an increase in the gene expression of nuclear factor erythroid 2-related factor 2 (NRF2), which is a major cell survival factor under stress conditions, the NRF2-regulated antioxidant response gene heme oxygenase-1 (*HO-1*) [84], as well as the ROS-stimulated hypoxia inducible factor (HIF)-target *VEGFA* [85] (Figure 7A–C). Treatment with catecholamines up-regulated *HO-1* and *VEGFA* mRNA in Jc1-infected cells too. These findings are in accordance with previous data supporting that exogenously provided dopamine leads to oxidative stress responses evidenced by an increased expression of *NRF2*, *HO-1* and hypoxia inducible factor-1α in human non-neuronal cells [86,87]. Viral infection also increased the expression of the antioxidant genes *NRF2* and *HO-1*, as well as of *VEGFA*, as shown by comparing mock-treated infected to mock-infected cells (Figure 7A,B), along with already published reports [88,89,90,91]. 

Then, as cells by utilizing reduced glutathione (GSH) diminish levels of H_2_O_2_ and ROS [92], we evaluated a possible role of GSH in HCV replication under treatment with dopamine. Huh7.5 cells were inoculated with JcR2a or Jc1 for 4 h and then treated or not with DA in the presence or absence of GSH, using concentrations that do not impact cell growth (Figure S3A,I), but influence oxidative stress-related gene expression (Figure 7A,B,D). Glutathione alone enhanced HCV replication whereas, in combination with dopamine, it diminished the negative impact of dopamine on HCV replication (Figure 7E,F) and in parallel, decreased the expression of oxidative stress-related genes (Figure 7D). Accordingly, H_2_O_2_ had a negative effect on Jc1 replication (i.e., negative and positive strand RNA amounts) (Figure S6A), while it induced the antioxidant response (Figure S6B). These results suggest that the cellular redox homeostasis mediates at least part of the role of the catecholamine biosynthesis and metabolism on HCV replication.

### 3.8. HCV Regulates the Expression of Catecholamine Biosynthesis Pathway-Related Enzymes

**Figure 8:**
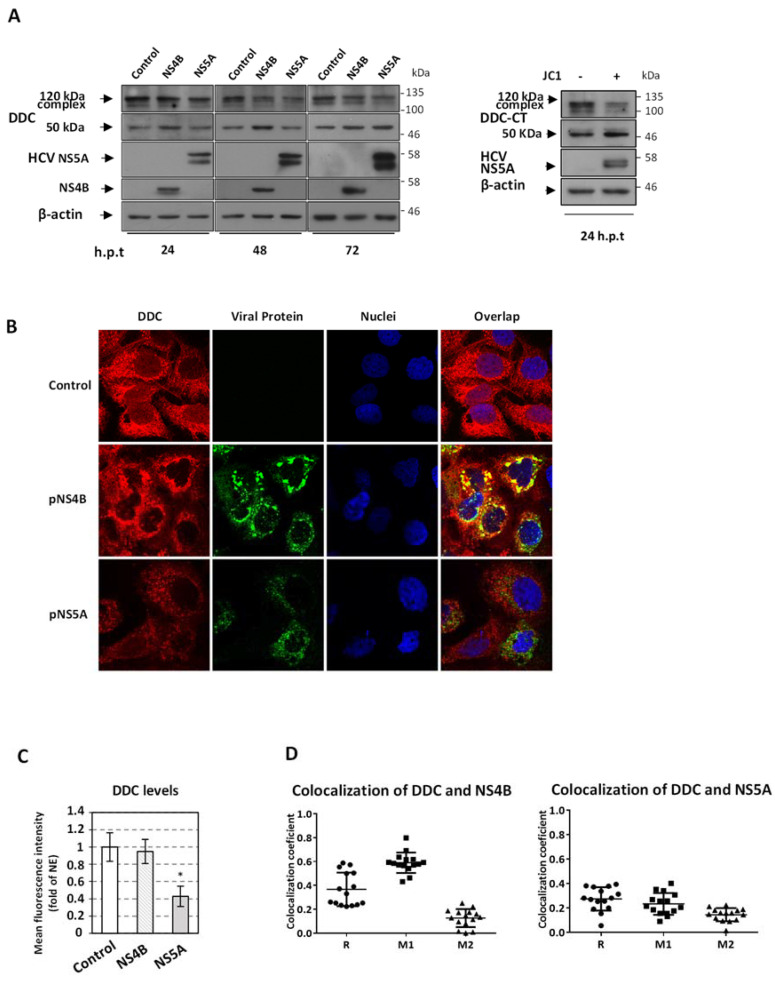
Effect of HCV protein expression on DDC levels. (**A**–**C**) Huh7-Lunet cells were electroporated with the NS4B (pEGFP-NS4B) or NS5A (pcDNA3-NS5A) expressing plasmid or with the pcDNA3 empty vector (control) and lysed at the indicated h.p.t. (**A**) Western blot analysis using anti-DDC, anti-NS4B and anti-NS5A antibodies. β-actin was used as loading control. In each time point, the overexpressed protein is mentioned above. A representative experiment of three independent repetitions is shown. (**B**) Immunofluorescence of the above, 48 h post-electroporated cells followed by confocal microscopy. Analysis of DDC (red) was performed using the rabbit polyclonal anti-DDC antibody, followed by confocal microscopy. HCV NS5A protein was stained with anti-NS5A antibody (green). HCV NS4B was detected through GFP fluorescence (green). Nuclei were stained with Hoechst 33,258 (blue). On the right, merged images of the green, red and blue fluorescence are shown. Bar, 20 μΜ. (**C**) Fold difference of mean DDC fluorescence intensity per cell, between cells electroporated with NS4B or NS5A and control cells, which was set as one. Error bars indicate standard deviations. * *p* < 0.001 vs. Control. (**D**) Colocalization coefficients between DDC and viral proteins. Graphical representation of the Pearson correlation coefficient (R) and Manders’ colocalization coefficient (M1, M2) values between DDC and NS4B (left) or NS5A (right). Each spot represents a single analyzed infected cell. Bars represent mean values obtained from three experiments (~30 analyzed cells/experiment).

Based on the negative regulation of HCV replication exerted by the components of the catecholamine biosynthetic and metabolic pathway and on the effect of HCV on DDC expression, we investigated whether HCV, in turn, alters the expression of other enzymes of the pathway and examined further how HCV affects DDC protein. Concerning the latter, we aimed to elucidate the mechanism(s) mediating the down-regulation of DDC protein by HCV infection [7]. This includes the reduction of its total intracellular levels, detected in IF, as well as an accumulation of the ~50 kDa DDC monomer with a concomitant reduction of a ~120 kDa DDC immunoreactive SDS-resistant protein, which is possibly the dimeric catalytically active form of the protein or a yet unknown DDC isoform species [7,8]. As the subgenomic HCV JFH1 replicon, which expresses only the non-structural viral proteins, exerts the same effect on DDC as the full-length virus [7], we examined the role of individual non-structural proteins in modifying DDC levels. More specifically, we overexpressed NS4B and NS5A in Huh7-Lunet cells and analyzed their effects on DDC. These two proteins were selected as they are known to interact with multiple host proteins and modulate viral replication [93]. Western blot analysis showed that, similarly to the HCV virus and subgenomic replicon [7], NS4B caused an accumulation of the ~50 kDa DDC monomer and a reduction of the ~120 kDa DDC immunoreactive species (Figure 8A). NS5A had the same phenotype only at the late time-point of 72 h post transfection, while it reduced the total DDC levels at earlier time-points (Figure 8A). In agreement with the Western blot results, NS5A appeared to reduce DDC protein levels also in IF (Figure 8B,C). Concerning its subcellular distribution, DDC did not colocalize significantly with NS5A (Pearson’s correlation coefficient: R = 0.28 ± 0.10, Manders’ colocalization coefficient: M1 = 0.23 ± 0.09), which is in consistence with the previously observed exclusion of DDC from the HCV replication sites [7] and (only partially) colocalized with NS4B (R = 0.37 ± 0.14, M1 = 0.59 ± 0.08) (Figure 8D). However, both viral proteins failed to affect the transcription of DDC up to 72 h post-transfection (Figure S4E). These results suggest that both viral proteins contribute to the phenotype of the virus-mediated negative regulation of DDC protein and that this phenotype is unrelated with the levels of expression of DDC.

**Figure 9:**
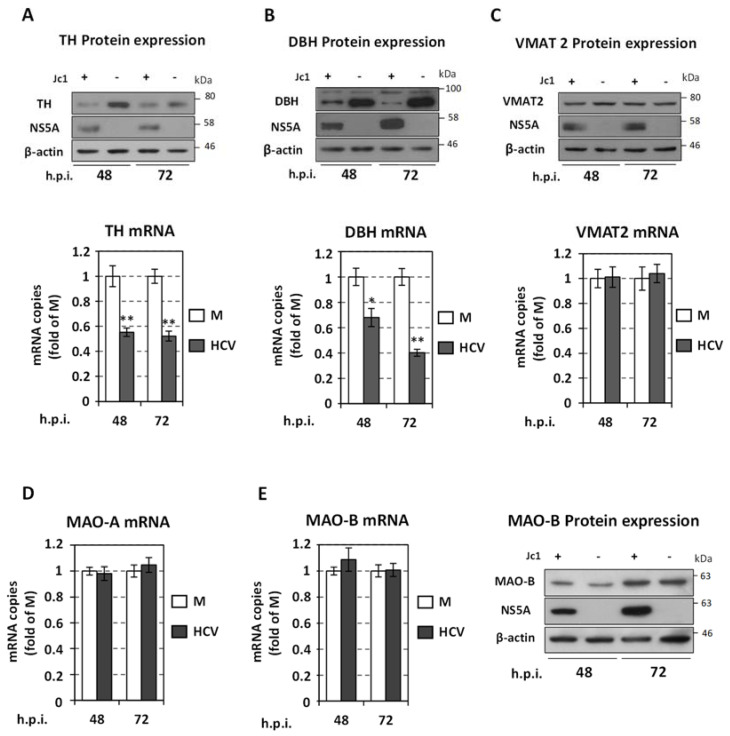
Effect of HCV infection on TH, DBH, VMAT2 and MAO levels. Huh7.5 cells were inoculated with Jc1 (MOI = 1), or mock-infected (M) and further cultured for the indicated h.p.i. (**A**–**C** upper and **E** right panels). Western blot analysis using anti-TH (**A**), anti-DBH (**B**), anti-VMAT2 (**C**) or anti MAO-B (**E**) antibodies. Viral infection was confirmed using anti-NS5A antibody. Mock-infected cells were used in parallel and are symbolized as Jc1 (−). β-actin was used as loading control. A representative experiment of three independent repetitions is shown. (**A**–**C** lower panel, **D**,**E** left panel) RT-qPCR analysis of TH, DBH, VMAT2, MAO-A and MAO-B mRNA levels normalized to YWHAZ mRNA. Values are expressed relative to the ones derived from mock-infected (M) cells, at each time-point. Bars represent mean values from three independent experiments in triplicate. Error bars indicate standard deviations. * *p* < 0.01, ** *p* < 0.001 vs. Control.

Then, we investigated the impact of HCV infection on the expression levels of enzymes other than DDC in the monoamine biosynthetic and metabolic pathway. We observed that Jc1 infection in Huh7.5 cells suppressed both protein and mRNA levels of TH (Figure 9A), which functions upstream of DDC by synthesizing L-Dopa. Similarly, a significant negative effect exerted by HCV was observed in both the protein and mRNA levels of DBH (Figure 9B), which converts DA to NE. Based on our aforementioned finding that inhibition of DBH does not impact HCV (Figure 4A–C), the negative effect of HCV on DBH might be indirect and result from the HCV-mediated down-regulation of the upstream biosynthetic enzyme DDC. Indeed, under conditions that DDC protein levels are reduced, as in the case of DDC silencing, a concomitant decrease of DBH levels occurs (Figure S1E). Moreover, the levels of the monoamine transporter VMAT2 (Figure 9C) were not altered during infection. In consistence with the tissue specificity of VMAT1 expression [18,94], its mRNA and protein were not detected in Huh7.5 cells (Figure S7). Finally, viral infection did not affect monoamine oxidase MAO-A/B mRNA amounts (Figure 9D,E), while it increased MAO-B at the protein level (Figure 9E).

In total, these data highlight an interesting bidirectional relationship between HCV replication and catecholamine biosynthesis in hepatocytes, unraveling for the first time the role of this pathway in viral infections.

## 4. Discussion

In this study, we report for the first time that the biosynthesis and uptake of catecholamines restrict the replication of HCV in hepatocytes. This effect on HCV is strongly related with oxidative stress that is generated by the auto-oxidation of catecholamines in the cell cytosol. Indeed, all treatments that are expected to enhance the levels of catecholamines in the cell cytosol or inhibit catecholamine deamination induced an antioxidant cell response, and at the same time, down-regulated the replication of HCV. Vice versa, the use of antioxidants or treatments that lower the levels of catecholamines in the cell cytosol positively affected the replication of HCV. To counteract the effect of catecholamines, HCV has developed ways to reduce at least their synthesis in the cell. Actually, our results showed that HCV infection, apart from the already reported effects on the levels of DDC mRNA and protein, caused the down-regulation of TH that encodes the rate-limiting enzyme of catecholamine biosynthesis, and reduced the levels of DBH mRNA and protein, although chemical inhibition of DBH activity seemed not to affect the virus replication in hepatocytes. Moreover, viral replication increased the levels of the MAO-B enzyme that degrades catecholamines through deamination and limits their auto-oxidation. In parallel, our results showed that the effect of HCV on DDC protein can be partially reproduced by the sole expression of NS4B viral protein, while the expression of NS5A protein negatively affected the total levels of DDC similarly to viral infection.

Previous studies have reported the dysfunction of the dopamine and serotonin signaling pathways in the brain of HCV patients, correlating viral infection with neurological manifestations [95,96]. While the role of DDC expression regulation in the periphery remains largely unknown, novel research findings demonstrated the involvement of DDC in the apoptotic cell death of both neuronal and non-neuronal cells [10]. Previous studies from our group have also indicated the physical and functional association of DDC with PI3K, the kinase that phosphorylates AKT, regulating cell survival [11]. Moreover, our recent report was the first to associate HCV infection with the down-regulation of the catecholamine biosynthetic pathway in the liver, as data from HCV-infected cells and DDC overexpression or chemical inhibition studies in cultured hepatocytes had shown the existence of a bidirectional negative relationship between HCV replication and DDC mRNA protein expression, dependent at least on the association of DDC with PI3K [7,11].

In the present study, we confirmed the negative effect of DDC on HCV by performing *DDC* gene silencing in infectious and subgenomic replicon systems and verified that this effect is exerted at the level of viral RNA replication. Concerning the underlying mechanism of the observed influence of DDC on HCV, apart from the previously reported PI3K/AKT signaling-related mechanism [7,11], our results indicated that HCV is also suppressed by the intracellular processing of DDC substrates into products, as both L-Dopa and 5-HTP had a negative effect on HCV replication, which was significantly attenuated upon *DDC* silencing. Thus, in accordance with our findings from the treatment with the DDC inhibitor carbidopa [7], these data revealed the importance of the biosynthetic role of DDC on HCV replication. Interestingly, the application of L-Dopa positively affected the transcription of DDC (Figure S4F) that putatively contributed as well to the effect on HCV. In addition to their biosynthesis, DDC products dopamine (DA) and serotonin (5-HT) also exerted a suppressive effect on HCV probably through their uptake, as their exogenous application in the cells was shown to reduce viral RNA and protein levels, while the use of the D2 dopamine receptor antagonist PCZ failed to reverse their effect on HCV. The latter suggested that the antiviral activity of the DDC biosynthetic pathway on HCV is not due to the autocrine/paracrine activation of the dopamine receptor D2, which has a well-characterized function in the liver, in inhibiting proliferation and migration and promoting apoptosis of HCC cells [63,64] and in regulating the detoxification function of hepatocytes [65]. The importance of the catecholamine uptake on HCV is also suggested by the results of treatment with the VMAT-specific, uptake and storage inhibitor reserpine [66,67,68,69,70,71,72,73]. Reserpine negatively affected the expression of OCT1, which encodes the major catecholamine transporter in hepatocytes [25,26], in parallel with the abrogation of the dopamine import in the cell. Accordingly, reserpine strongly induced the replication of HCV, while it abrogated the negative effect of the exogenously applied dopamine on the virus. Also, reserpine caused a down-regulation in the expression of DDC and DBH in infected cells, which is expected due to the higher levels of viral replication. Apart from the virus replication induction, a possible inhibition of the uptake of the cell culture medium containing L-Dopa, which is also a substrate of OCT1 [97], could account for the observed effect on these proteins. This is strengthened by the fact that the exogenously applied L-Dopa up-regulates DDC.

We further validated the negative relation of the catecholamine biosynthetic pathway with HCV infection by examining the implication of other proteins of the catecholamine biosynthesis (TH, DBH) and metabolism (MAO-A/B), applying their enzymatic products or using inhibitors of their activities.

Specifically, we focused first on norepinephrine (NE) and its biosynthetic enzyme DBH that synthesizes NE from DA inside the monoamine storage vesicles. Combined, our data, with the use of the DBH inhibitor nepicastat [75] and the catecholamine uptake and storage inhibitor reserpine, suggested that viral replication is suppressed by the uptake and presence of NE in the cell cytosol, however this cannot occur when NE is restricted inside the cytoplasmic vesicles. In contrast to DBH, which was shown to be expressed in Huh7.5 cells, in consistence with previous data from liver tissue [16], we did not detect, and thus did not study PNMT that converts norepinephrine to epinephrine, and this is in agreement with previous studies showing that the biosynthesis of epinephrine in the liver is accomplished by a non-specific methyltransferase and not PNMT [17].

The negative impact of catecholamines and serotonin on HCV was further supported by the use of the MAO inhibitors phenelzine and clorgyline, which suppress the oxidative deamination (degradation) of monoamines and thus increase their intracellular levels and autooxidation [12,13].

Our data showing that various steps of the biosynthetic and metabolic route of serotonin and catecholamines down-regulate HCV replication, were further supported by the negative effect of PKA and PKC inducers forskolin and PMA on the virus. These kinases up-regulate the biosynthetic enzymes DDC and TH [12,78] and the metabolic enzyme MAO [79].

Catecholamines that are not stored inside cytoplasmic vesicles or deaminated by MAO enzymes [12,13] can be auto-oxidated in the cell cytosol, producing toxic quinones that also generate ROS. This leads to oxidative stress, as has been observed by the induction of the expression of antioxidant genes (*NRF2*, *HO-1*) and the ROS stimulated HIF [86,87]. Our data confirmed that catecholamines induced the oxidative stress response gene expression (*NRF2*, *HO-1* and *VEGFA*) in both infected and mock-infected Huh7.5 cells, as well as verified the negative role of MAO activity in this procedure. Interestingly, by the use of reduced GSH, we revealed that the down-regulation of HCV replication by catecholamines is strongly dependent by the catecholamine-related alterations on cell redox homeostasis. These results are in agreement with previous studies that suggest a negative effect of ROS on HCV replication [81,82,83]. Our results also indicated that the HCV infection itself also induced the expression of oxidative stress-related genes. This either could be attributed to a real oxidative stress generated by the virus replication [88,89,90,91] or could be part of a mechanism employed by the virus in order to shift the cell redox homeostasis to a more reduced environment aiming to favor viral replication. Evidence of the latter could be the results acquired by the sole use of GSH that positively affected the HCV replication.

Apart from the oxidative stress induced by catecholamines, which appears to control their antiviral effect, the implication of novel roles of these molecules remains to be elucidated. Recent studies showed that dopamine and serotonin covalently bind to histones regulating gene expression through a process called dopaminylation [98] and serotonylation [99], respectively. Moreover, the interaction of HCV with the monoamine biosynthetic and metabolic pathway may be related to the function of its products in cell proliferation and apoptosis of hepatocytes [28,29,30,31,32,33,34,35,36].

Furthermore, the ability of HCV to suppress the expression of the catecholamine biosynthetic enzymes DDC [7], TH and DBH, as well as to increase the levels of catecholamine degradation enzyme MAO-B, indicates that the virus counteracts this pathway for optimal replication. However, the virus did not alter the expression of the monoamine storage enzyme VMAT2, even though the inhibition of its activity impacted viral replication. Taking into account that ΤH is the rate-limiting enzyme of catecholamine biosynthesis [100], while DDC and DBH become rate-limiting under specific cases [78,101], it is possible that targeting the biosynthetic and metabolic enzymes is an efficient mechanism for the virus to resist the antiviral action of catecholamines.

Concerning DDC, we have previously shown that HCV mediates an accumulation of the ~50 kDa DDC monomer with a concomitant reduction of a ~120 kDa DDC complex, possibly corresponding to the dimeric catalytically active form of the protein [7,8]. Moreover, HCV was observed to reduce the total DDC intracellular levels in IF [7]. Towards the elucidation of the virus-mediated DDC down-regulation, here we showed that the non-structural viral proteins NS4B and NS5A are both implicated, but through different mechanisms. Specifically, NS4B simulates the effect of HCV on the accumulation of the 50 kDa DDC monomer and the decrease of the 120 kDa complex. However, in contrast to HCV infection, where DDC does not colocalize with the viral replication sites [7], DDC was partially colocalized with NS4B upon its overexpression. NS5A reduced total DDC protein levels and, late after transfection, when higher levels of the viral protein have been expressed, accumulated the 50 kDa monomer. Moreover, NS5A did not colocalize with DDC in the endoplasmic reticulum, in agreement with the infection assays [7]. However, although HCV reduces *DDC* mRNA [7], neither NS4B nor NS5A affected *DDC* mRNA levels, which suggests that they regulate DDC protein through post-transcriptional mechanisms. The colocalization of DDC subunits with NS4B may indicate an additional anti-viral mechanism which extends beyond that of dopamine biosynthesis. Based on this finding and on our earlier observation that viral infection disturbs DDC-PI3K complex formation [7,11], a possible NS4B-DDC interaction could hinder the collaboration of NS4B with PI3K (CLASS III VSP34) to facilitate viral replication [102]. In addition, NS5A may regulate DDC levels by promoting its degradation, as DDC has been implicated in programmed cell death [10] and NS5A is known to counteract apoptosis in infected cells by inducing the degradation of apoptosis-related proteins, such as p53 [103] and IP3R3 [104].

The research described in the manuscript has been limited in cell-culture based infectious models and thus an in vivo correlation between HCV infection and the expression of catecholamine biosynthetic pathway enzymes other than DDC remains to be studied. Moreover, any implication on HCV replication exerted by the function of catecholamines and serotonin in gene expression or in cell proliferation and apoptosis should be addressed.

The present study revealed an intricate bidirectional relationship between HCV and key molecules of the catecholamine and serotonin pathway, unraveling new determinants of viral replication and contributing to the increasing evidence for the role of bioactive amines in peripheral tissues.

## Figures and Tables

**Table 1:** Priming oligonucleotides used for RT-qPCR analysis.

Target	Orientation	Sequence (5′ - 3′)
JFH1-276-F	Forward	GGCCTTGTGGTACTGCCTGATA
JFH1-354-R	Reverse	GGATTTGTGCTCATGGTGCA
*DDC*	Forward	GAACAGACTTAACGGGAGCCTTT
	Reverse	AATGCCGGTAGTCAGTGATAAGC
*TH*	Forward	GGAAGGCCGTGCTAAACCT
	Reverse	GGATTTTGGCTTCAAACGTCTC
*DBH*	Forward	GCCTTCATCCTCACTGGCTACT
	Reverse	CAGCACTGTGACCACCTTTCTC
*MAOA*	Forward	GGGCTGCTACACGGCCTACT
	Reverse	GACCTCCCTAGCTGCTCGTTCT
*MAOB*	Forward	GGAGCCAGTGCATTATGAAGA
	Reverse	GCCTGCAAAGTAAATCCTGTC
*VMAT2*	Forward	CGGATGTGGCATTTTGTATGG
	Reverse	TTCTTCTTTGGCAGGTGGACTTC
*OCT1*	Forward	CACCCCCTTCATAGTCTTCAG
	Reverse	GCCCAACACCGCAAACAAAAT
*NRF2*	Forward	TGAGCAAGTTTGGGAGGAGC
	Reverse	GGCTTCTGGACTTGGAACCAT
*HO-1*	Forward	ATGACACCAAGGACCAGAGC
	Reverse	GTGTAAGGACCCATCGGAGA
*VEGFA*	Forward	CTTGCCTTGCTGCTCTAC
	Reverse	TGGCTTGAAGATGTACTCG
*YWHAZ*	Forward	GCTGGTGATGACAAGAAAGG
	Reverse	GGATGTGTTGGTTGCATTTCCT

## Data Availability

All relevant data are within the manuscript and its Supporting information files.

## Supplementary Materials

The following are available online at https://www.mdpi.com/article/10.3390/v13112139/s1, Supplementary Materials and Methods, Figure S1: Virus replication kinetics and *DDC* silencing, Figure S2: Effect of L-Dopa and 5-HTP on intracellular ATP levels and HCV replication, Figure S3: Effect of catecholamine pathway-associated products, biosynthetic enzymes and transporters inhibitors or inducers on intracellular ATP levels, Figure S4: DDC gene expression in response to catecholamine pathway products and substrates, VMAT-specific inhibitor reserpine and HCV protein expression, Figure S5: Effect of catecholamine pathway products and inhibitors of the pathway on HCV genotype 1b replicon, Figure S6: Effect of H_2_O_2_ on viral replication, Figure S7: Investigation of VMAT1 presence on HCV infected hepatocytes.
