# Supplementary material for: Association of Hepatitis C Virus Replication with the Catecholamine Biosynthetic Pathway
Source: Viruses. 2021 Oct 23;13(11):2139. doi: 10.3390/v13112139 (PMC8624100; doi:10.3390/v13112139)
Supplement: Supplementary file 1 [file viruses-13-02139-s001.zip › viruses-1422009-supplementary.pdf]

viruses

MDPI

Supplementary Materials

Association of Hepatitis C Virus Replication with the Catecholamine Biosynthetic Pathway

George Mpekoulis [1,†], Vassilina Tsopela [1,†], Georgios Panos [1,†], Vasileios Siozos [1,†], Katerina I. Kalliampakou [1], Efseveia Frakolaki [1,‡], Constantinos D. Sideris [1], Alice G. Vassiliou [2], Diamantis C. Sideris [3], Dido Vassilacopoulou [3] and Niki Vassilaki [1,*]

[1] Laboratory of Molecular Virology, Hellenic Pasteur Institute, 11521 Athens, Greece; g.mpekoulis@pasteur.gr (G.M.); vas.tsopela@gmail.com (V.T.); panos.georgios.bio@gmail.com (G.P.); basilis.sz@hotmail.com (V.S.); kalliamp@yahoo.gr (K.I.K.); sevif@pasteur.gr (E.F.); con97sider@gmail.com (C.D.S.)
[2] GP Livanos and M Simou Laboratories, 1st Department of Critical Care Medicine & Pulmonary Services, School of Medicine, National and Kapodistrian University of Athens, Evangelismos Hospital, 10676 Athens, Greece; alvass75@gmail.com
[3] Section of Biochemistry and Molecular Biology, Faculty of Biology, National and Kapodistrian University of Athens, 15701 Athens, Greece; dsideris@biol.uoa.gr (D.C.S.); didovass@biol.uoa.gr (D.V.)
* Correspondence: nikiv@pasteur.gr; Tel.: +30-210-647-8875
† Equal contribution.
‡ Deceased.

Citation: Mpekoulis, G.; Tsopela, V.; Panos, G.; Siozos, V.; Kalliampakou, K.I.; Frakolaki, E.; Sideris, C.D.; Vassiliou, A.G.; Sideris, D.C.; Vassilacopoulou, D.; et al. Association of Hepatitis C Virus Replication with the Catecholamine Biosynthetic Pathway. *Viruses* **2021**, *13*, 2139. https://doi.org/10.3390/v13112139

Academic Editors: Ioannis Karakasiliotis, Apostolos Beloukas and Serafeim Chaintoutis

Received: 29 September 2021
Accepted: 19 October 2021
Published: 23 October 2021

Publisher's Note: MDPI stays neutral with regard to jurisdictional claims in published maps and institutional affiliations.

Copyright: © 2021 by the authors. Licensee MDPI, Basel, Switzerland. This article is an open access article distributed under the terms and conditions of the Creative Commons Attribution (CC BY) license (http://creativecommons.org/licenses/by/4.0/).

1. Supplementary Materials and Methods

1.1. Cell Culture

Huh5-2 stable cell line harboring the subgenomic reporter replicon of genotype 1b (Con1) [50]. This replicon is composed of the HCV 5′ UTR possessing an internal ribosome entry site (IRES) that directs the expression of a firefly luciferase–ubiquitin–neomycin phosphotransferase fusion protein (luc-ubi-neo). The expression of the luc-ubi-neo fusion protein enables the selection for cells containing the replicon using neomycin/G418 and the assessment of viral RNA replication by measuring the activity of the reporter protein firefly luciferase (F-Luc).

1.2. Antibodies and Chemicals

Hydrogen peroxide (H_2O_2) was purchased by Sigma-Aldrich (Taufkirchen, Germany). VMAT1 mouse monoclonal antibody (clone G-12; Santa Cruz Biotechnology) was used at a dilution of 1:500.

1.3. RNA Quantification by Reverse Transcription-Quantitative PCR (RT-qPCR)

For HCV positive and negative strand RNA quantitation, reverse transcription (RT) reactions were performed using HCV-specific primers JFH1-354R and JFH1-276F, respectively, as well as the primer YWHAZ-R (Table1), specific for the housekeeping gene 14-3-3-zeta polypeptide (YWHAZ) as an internal control (3.5 pmol/μL of each primer). Real-time quantitative PCR was performed using Luna® Universal qPCR Master Mix [New England Biolabs, Inc. Ipswich, MA, USA] as well as primer pairs specific for the HCV IRES (JFH1-276F and JFH1-354R). YWHAZ housekeeping gene was used as a normalization control in all qPCR reactions.

1.4. Plasmid Constructs

The subgenomic replicon construct pFK_i389LucNS3-3'_dg_JFH (with a Firefly luciferase gene), based on the HCV JFH1 strain, has been described previously [104].

1.5. Preparation and Titration of Virus Stocks and Infection Assays

HCV virus stocks were generated in Huh7-Lunet cells as described elsewhere [57] and used to infect naive Huh7.5 cells. HCV was titrated as described elsewhere [58], using the JFH1 NS5A-specific mouse monoclonal antibody 9E10 [58]. Infectivity titers were expressed as the 50% tissue culture infective dose (TCID50)/mL.

1.6. Generation of stable Huh7.5 derived cell lines expressing shDDC or shControl RNA

Lentiviral particles were produced by lipofectamine transfection of 293T cells. Three plasmids were transfected via Lipofectamine 2000 (Invitrogen, Carlsbad, CA) according to the manufacturer's instructions, the vesicular stomatitis virus envelope glycoprotein expression construct pczVSV-G [105], the HIV-1 Gag-Pol expression construct pRCMVΔR8.74 [106] and the vector psi-LVRH1GP/shDDC (shDDC) or the respective negative control vector (shControl). Briefly, 293T cells were seeded at a density of 0.5 x 10^6 cells/well in a 6-cm diameter plate and 24 h later were transfected with the three plasmids at a ratio of 1:3:3 (2.14μg:6.42μg:6.42μg), respectively. The medium was replaced 8 h after transfection. Supernatants containing the lentiviral pseudoparticles that encode shDDC or only the negative control shControl, were harvested 48h later, cleared by passage through 0.45-μm pore sized filters and used to inoculate 4x10^4 Huh7.5 cells. 6h later supernatants were replaced with complete DMEM and transduced cells were selected by using medium supplemented with 2μg/ml puromycin at 48h post-inoculation and afterwards. *DDC* silencing was confirmed by measuring intracellular DDC mRNA and protein levels.

2. Supplementary Figures

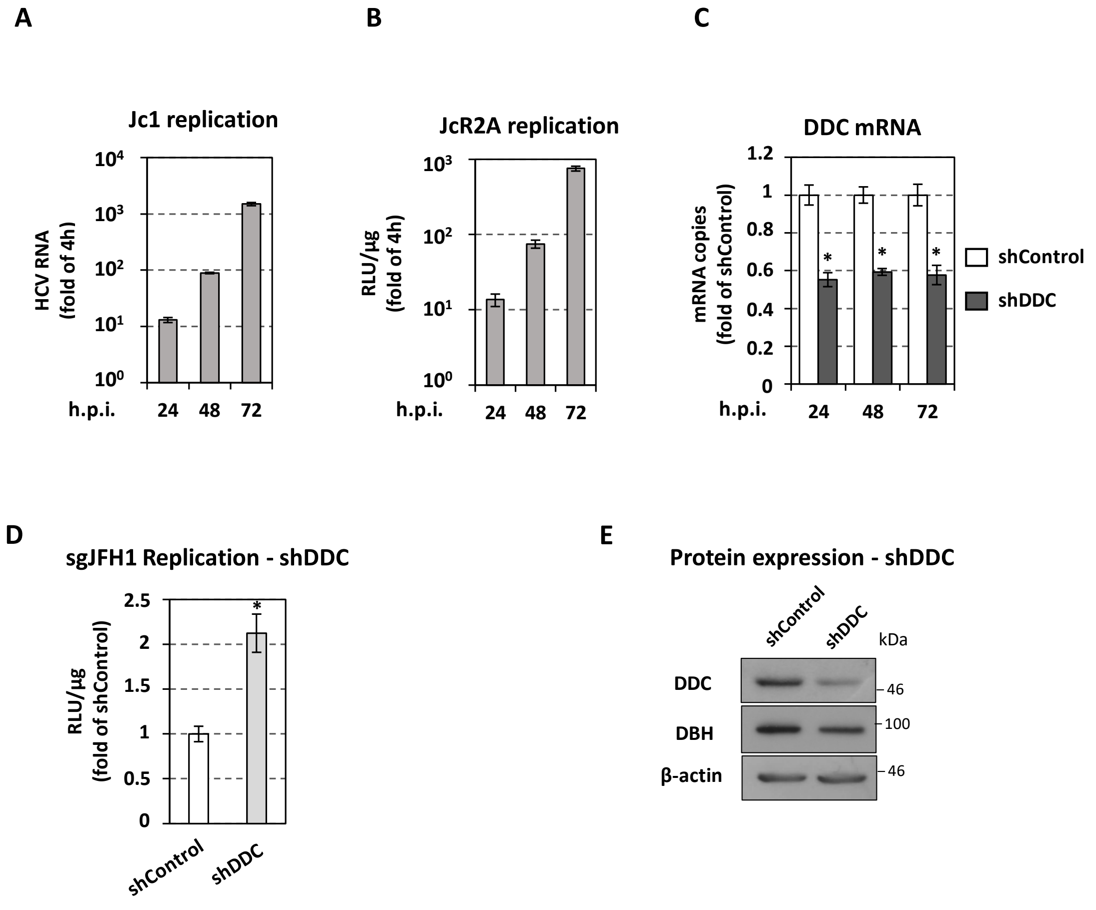

Figure S1. Virus replication kinetics and DDC silencing. (**A–C**) Huh7.5 cells were inoculated with Jc1 (MOI = 1) (**A**,**C**) or JcR2A (MOI = 0.5) (**B**) for 4 h. (**A**) Jc1 positive-strand RNA levels were quantified by RT-qPCR and YWHAZ mRNA was used for normalization. (**B**) Viral replication derived Renilla luciferase (R-Luc) activity was measured and expressed as relative light units (RLU) per μg of total protein amount. Values are expressed relatively to the ones obtained at 4 h. (**C**) Effect of DDC silencing on DDC mRNA levels. Huh7.5 cells were electroporated with psi-LVRH1GP/sh-DDC (shDDC) plasmid or the control vector (shControl), 24 h post-electroporation were infected with Jc1 and lysed at the indicated time points. DDC mRNA levels were determined by RT-qPCR and normalized to YWHAZ mRNA. Values from shControl cells were set to one at each time-point. (**D**) Effect of DDC silencing on HCV replication. Huh7.5 cells stably expressing shDDC or shControl were transfected with in vitro transcribed subgenomic reporter HCV JFH1 RNA (0.5 μg sgJFH1/ 0.7 x 10^4 cells) via Lipofectamine 2000 (Invitrogen, Carlsbad, CA) according to the manufacturer's instructions and further cultured for 72 h. Values are normalized to the ones obtained at 4 h and expressed as fold of shControl. Sg-JFH1 replication-derived R-Luc activity was quantified by chemiluminescence-based assay and expressed as RLU/μg of total protein amount. (**A–D**) Bars represent mean values from three independent experiments in triplicate. Error bars indicate standard deviations. Values from shControl cells were set to one. * $p < 0.001$ vs shControl. (**E**) Effect of DDC silencing to DBH protein levels. Western blot analysis was performed in lysates of Huh7.5 cells stably expressing shDDC or shControl, using anti-DBH, anti-DDC or anti-β-actin antibodies. β-actin was used as loading control. A representative experiment of three independent repetitions is shown.

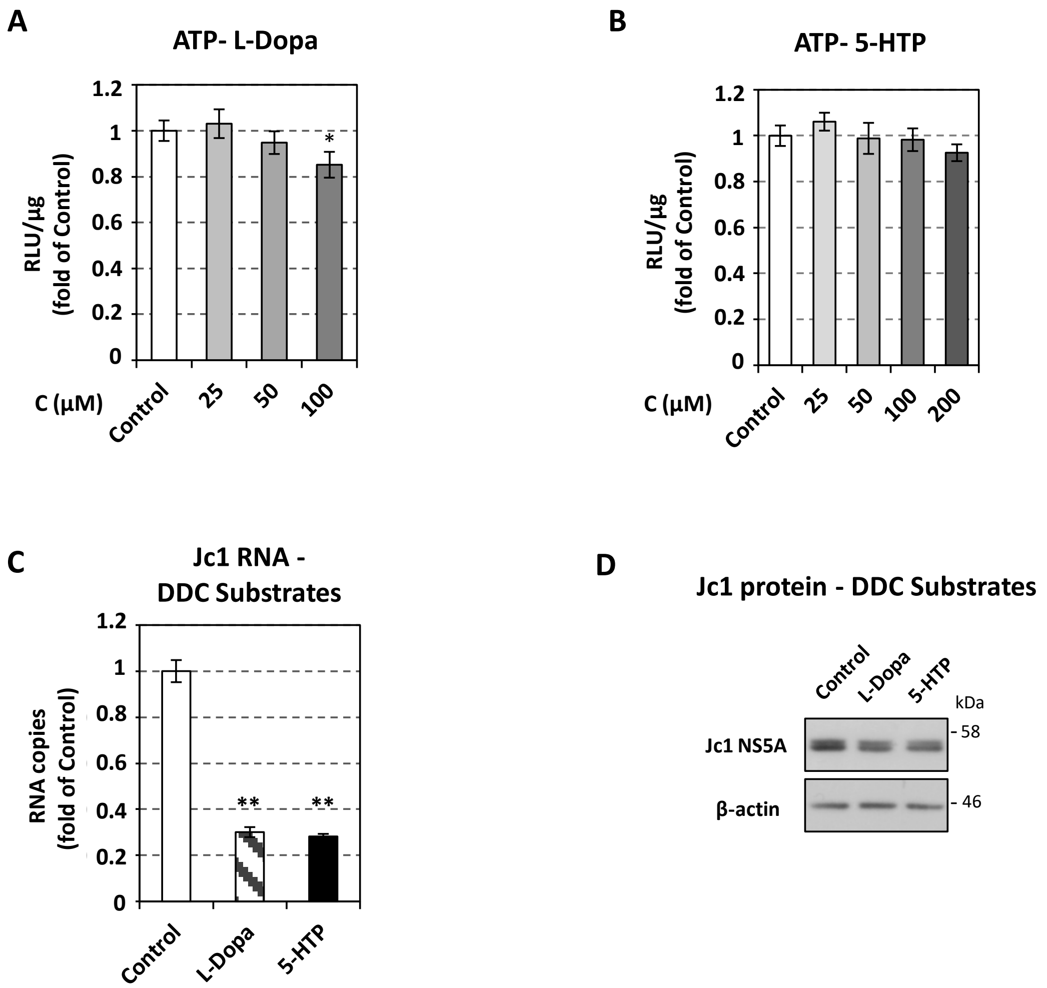

Figure S2. Effect of L-Dopa and 5-HTP on intracellular ATP levels and HCV replication. (**A**,**B**) Effect of different concentrations of the L-Dopa (**A**) and 5-HTP (**B**) on the intracellular ATP levels of Huh7.5 cells, as measured using a chemiluminescence-based assay and expressed as RLU/μg of total protein amount. Values from mock-treated (Control) cells were set to one. (**C**,**D**) Huh7.5 cells were inoculated with Jc1 (MOI = 1) for 4 h and subsequently treated for 72 h with 50 μM L-Dopa or 100 μM 5-HTP, or mock-treated (Control). (**C**) Jc1 positive-strand RNA levels were quantified by RT-qPCR and YWHAZ mRNA was used for normalization. Values from mock-treated (Control) cells were set to one. Bars represent mean values from three independent experiments in triplicate. Error bars indicate standard deviations. * $p < 0.05$, ** $p <$

0.001 vs Control. (**D**) Western blot analysis of HCV NS5A. β-actin was used as loading control. A representative experiment of three independent repetitions is shown.

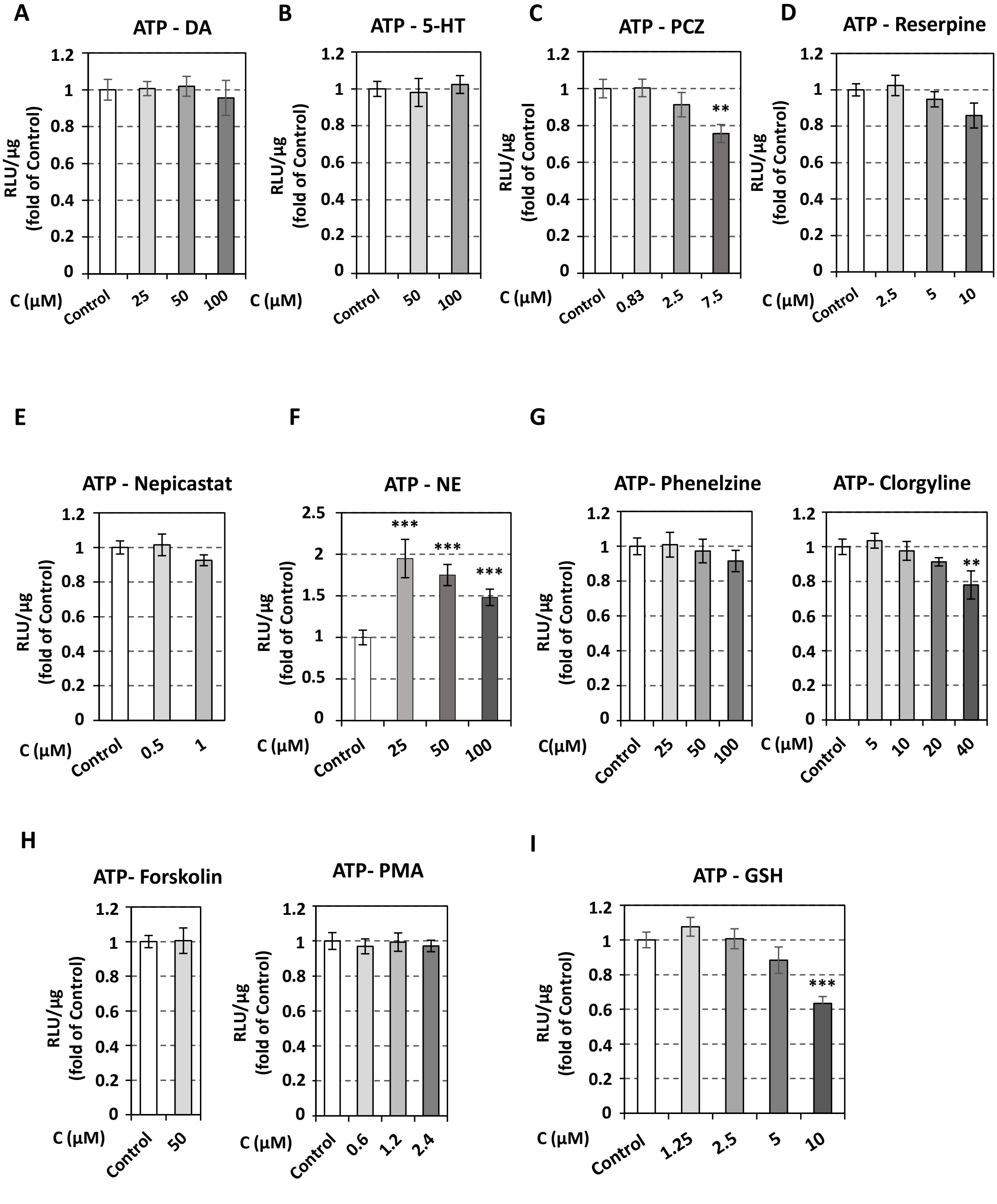

Figure S3. Effect of catecholamine pathway-associated products, biosynthetic enzymes and transporters inhibitors or inducers on intracellular ATP levels. Huh7.5 cells were treated for 72 h with the indicated concentrations of DA (**A**), 5-HT (**B**), PCZ (**C**), reserpine (**D**), nepicastat (**E**), NE (**F**), phenelzine, clorgylin (**G**), forscolin, PMA (**H**), GSH (**I**) or mock-treated (Control). Intracellular ATP levels were determined using a chemiluminescence-based assay and expressed as RLU/µg of total protein amount. Values from control cells were set to one. Bars represent mean values from three independent experiments in triplicates. Error bars indicate standard deviations. * $p < 0.05$, ** $p < 0.01$, *** $p < 0.001$ vs Control.

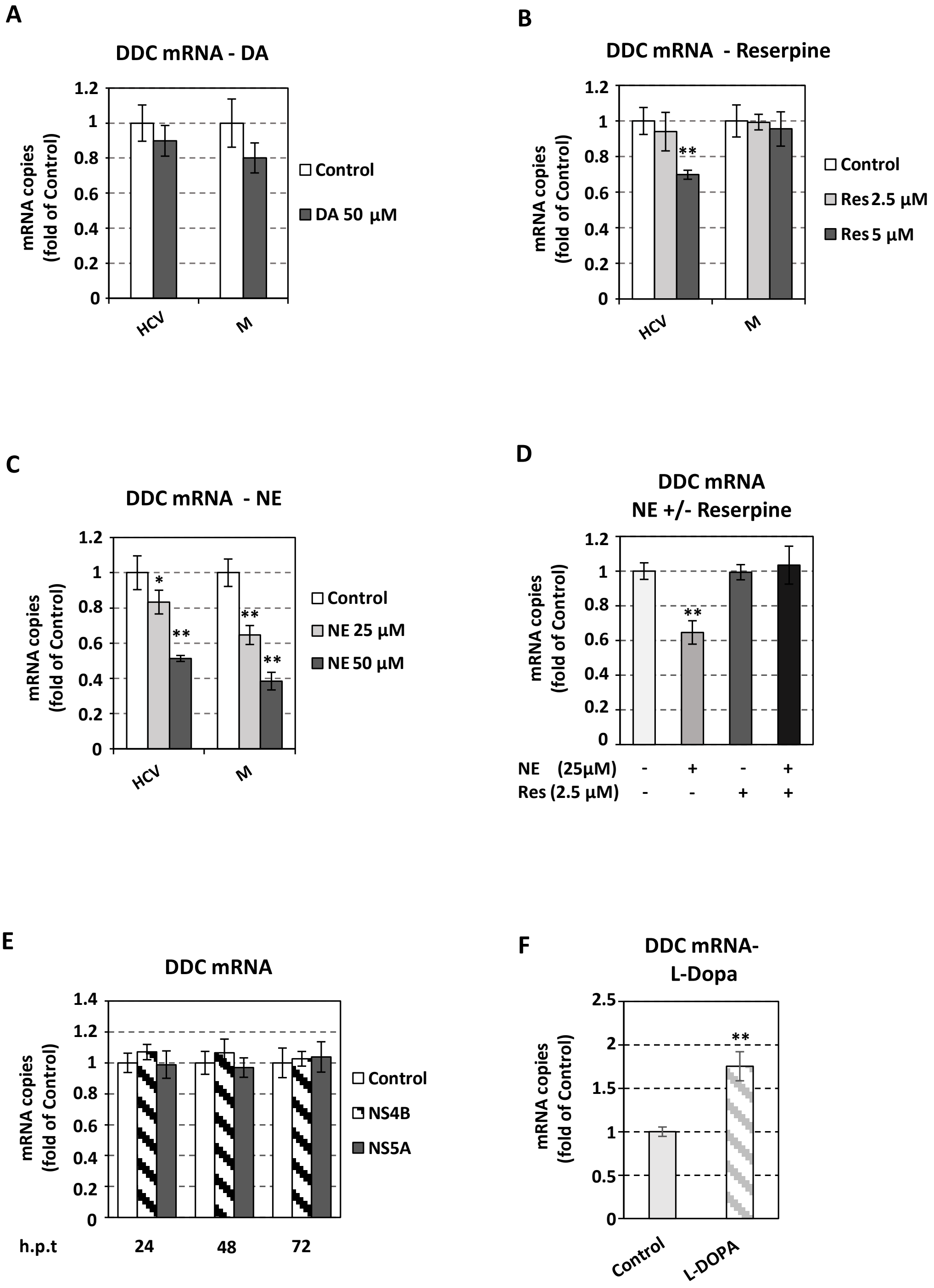

Figure S4. DDC gene expression in response to catecholamine pathway products and substrates, VMAT-specific inhibitor reserpine and HCV protein expression. (**A**–**C**) Huh7.5 cells were inoculated with JcR2a (MOI = 0.5) for 4 h, or mock-infected (M) and subsequently treated with different concentrations of DA or mock-treated (Control) for 72 h (**A**), reserpine (**B**) or NE (**C**). (**D**) Huh7.5 cells were treated with the indicated concentrations of NE and reserpine. (**A**–**D**) Values from Control cells were set to one. (**E**) Huh7-Lunet cells were electroporated with the NS4B (pEGFP-NS4B) or NS5A (pcDNA3-NS5A) expressing plasmid or with the pcDNA3 empty vector (Control) and lysed at the indicated h p.t. Values from control cells were set to one at each time point. (**F**) Huh 7.5 cells were treated with 50 µM L-Dopa, or mock-treated (Control) for 72 h. Values from Control cells were set to one. In all panels, DDC mRNA levels were quantified by RT-qPCR and normalized to YWHAZ mRNA. Bars represent mean values from three independent experiments in triplicate. Error bars indicate standard deviations. * $p < 0.01$, ** $p < 0.001$ vs Control.

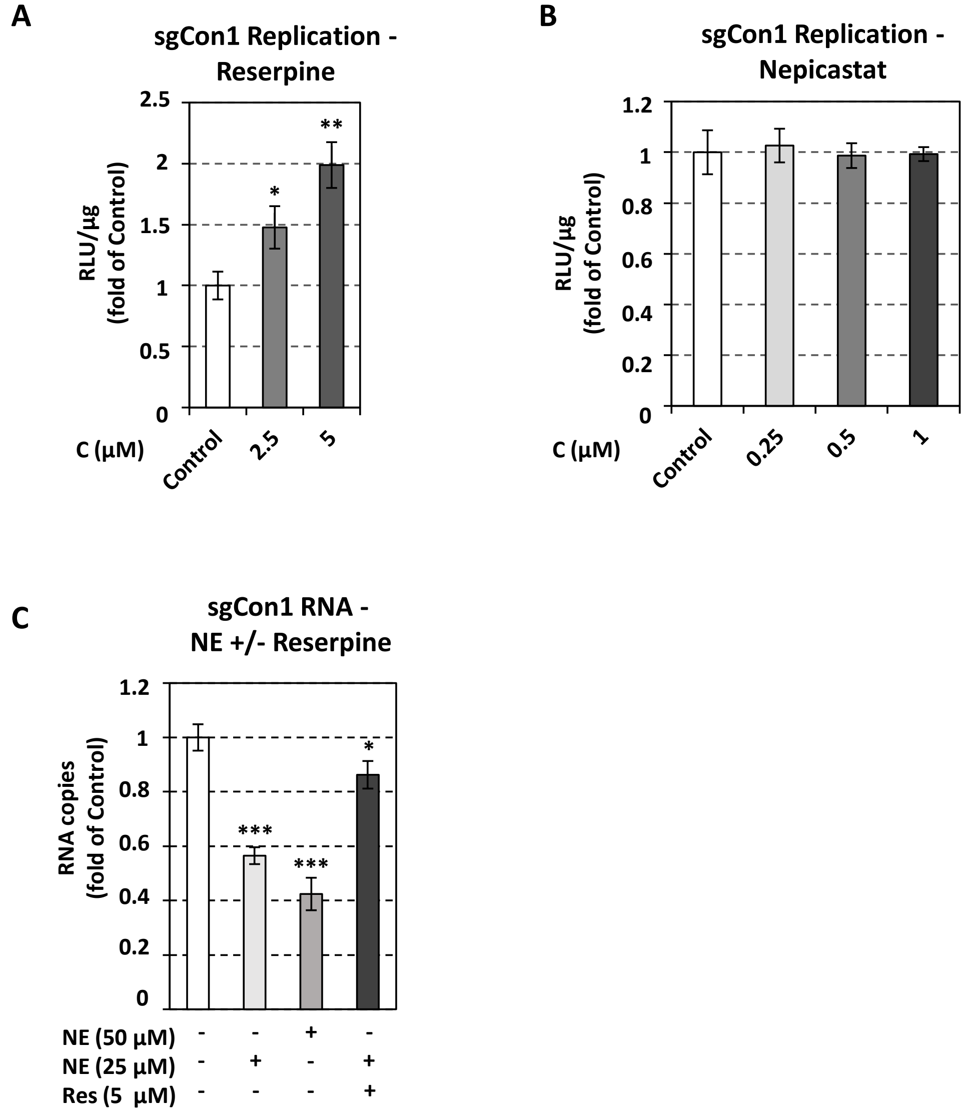

Figure S5. Effect of catecholamine pathway inhibitors and NE on HCV genotype 1b replicon. Huh5-2 cells, harboring HCV genotype 1b (Con1) subgenomic replicon, were treated for 72 h, with the indicated concentrations of reserpine (**A**,**C**), nepicastat (**B**), NE (**C**) or were mock-treated (Control). (**A**,**B**) Viral RNA replication-derived firefly luciferase activity was determined and expressed as RLU/µg of total protein. (**C**) Positive strand HCV RNA was quantified by RT-qPCR and normalized to the mRNA levels of the housekeeping gene YWHAZ. In all panels, values from control cells were set to one. Bars represent mean values from the independent experiments in triplicate. Error bars indicate standard deviations. * $p <$ 0.5, ** $p < 0.01$, *** $p < 0.001$ vs Control.

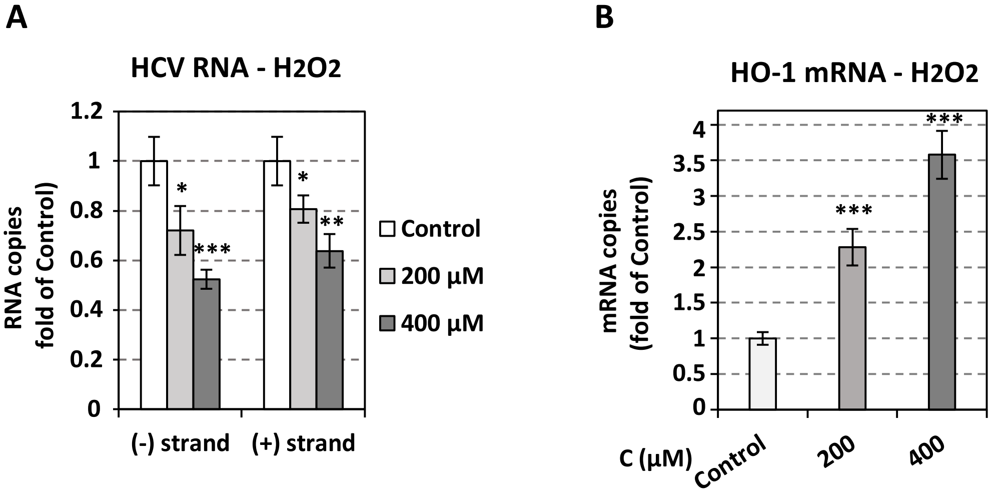

Figure S6. Effect of H_2O_2 on viral replication. Huh7.5 cells were inoculated with Jc1 (MOI = 1) for 4 h, or mock-infected (M) and subsequently treated or not (Control) for 72 h with different concentrations of H_2O_2. (**A**) Positive and negative strands of HCV RNA were quantified by RT-qPCR and normalized to the mRNA levels of the housekeeping gene YWHAZ. Values from infected mock-treated cells were set to one for each strand. (**B**) HO-1 mRNA levels were quantified by RT-qPCR and normalized to YWHAZ mRNA. Values from control cells were set to one. Bars represent mean values from three independent experiments in triplicate. Error bars indicate standard deviations. * $p < 0.05$, ** $p < 0.01$, *** $p < 0.001$ vs Control.

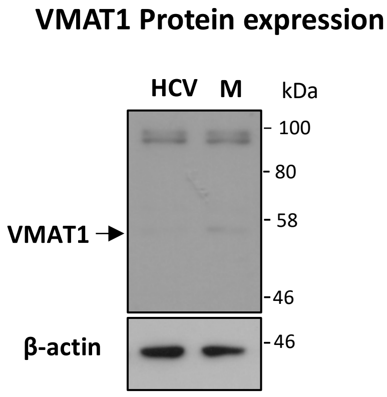

Figure S7. Investigation of VMAT1 presence on HCV infected hepatocytes. Huh7.5 cells were inoculated with Jc1 (MOI = 1) for 4 h or mock-infected and further incubated for 72 h. Western blot analysis was performed in lysates of Jc1-infected, or mock-infected cells using anti-VMAT1 and anti-β-actin antibodies, β-actin was used as loading control.

References

1. Vrolijk, J.M.; Kaul, A.; Hansen, B.E.; Lohmann, V.; Haagmans, B.L.; Schalm, S.W.; Bartenschlager, R. A replicon-based bioassay for the measurement of interferons in patients with chronic hepatitis C. *J. Virol. Methods* **2003**, *110*, 201–209, doi:10.1016/s0166-0934(03)00134-4.
57. Vassilaki, N.; Friebe, P.; Meuleman, P.; Kallis, S.; Kaul, A.; Paranhos-Baccala, G.; Leroux-Roels, G.; Mavromara, P.; Bartenschlager, R. Role of the hepatitis C virus core+1 open reading frame and core cis-acting RNA elements in viral RNA translation and replication. *J. Virol.* **2008**, *82*, 11503–11515, doi:10.1128/JVI.01640-08.
58. Lindenbach, B.D.; Evans, M.J.; Syder, A.J.; Wolk, B.; Tellinghuisen, T.L.; Liu, C.C.; Maruyama, T.; Hynes, R.O.; Burton, D.R.; McKeating, J.A.; et al. Complete replication of hepatitis C virus in cell culture. *Science* **2005**, *309*, 623–626, doi:10.1126/science.1114016.

104. Schaller, T.; Appel, N.; Koutsoudakis, G.; Kallis, S.; Lohmann, V.; Pietschmann, T.Bartenschlager, R. Analysis of hepatitis C virus superinfection exclusion by using novel fluorochrome gene-tagged viral genomes. *J Virol* **2007**, *81*, 4591–603. DOI: 10.1128/JVI.02144-06.
105. Kalajzic, I.; Stover, M.L.; Liu, P.; Kalajzic, Z.; Rowe, D.W.Lichtler, A.C. Use of VSV-G pseudotyped retroviral vectors to target murine osteoprogenitor cells. *Virology* **2001**, *284*, 37–45.
106. Dull, T.; Zufferey, R.; Kelly, M.; Mandel, R.J.; Nguyen, M.; Trono, D.Naldini, L. A third-generation lentivirus vector with a conditional packaging system. *J Virol* **1998**, *72*, 8463–8471.
